# Structure of endothelin ET_B_ receptor–G_i_ complex in a conformation stabilized by unique NPxxL motif

**DOI:** 10.1038/s42003-024-06905-z

**Published:** 2024-10-16

**Authors:** Kazutoshi Tani, Saori Maki-Yonekura, Ryo Kanno, Tatsuki Negami, Tasuku Hamaguchi, Malgorzata Hall, Akira Mizoguchi, Bruno M. Humbel, Tohru Terada, Koji Yonekura, Tomoko Doi

**Affiliations:** 1 Center for Computational Sciences, University of Tsukuba, 1-1-1 Tennodai, Tsukuba, Ibaraki, 305-8577 Japan; 2 Graduate School of Medicine, Mie University, 2–174 Edobashi Tsu, Mie, 514-8507 Japan; 3 RIKEN SPring-8 Center, 1-1-1, Kouto, Sayo, Hyogo, 679-5148 Japan; 4 Scientific Imaging Section, Research Support Division, Okinawa Institute of Science and Technology Graduate University (OIST), 1919-1, Tancha, Onna-son, Kunigami-gun, Okinawa, 904-0495 Japan; 5 Quantum Wave Microscopy Unit, Okinawa Institute of Science and Technology Graduate University (OIST), 1919-1, Tancha, Onna-son, Kunigami-gun, Okinawa, 904-0495 Japan; 6 Department of Biotechnology, Graduate School of Agricultural and Life Sciences, University of Tokyo, Bunkyo-ku, Tokyo, 113-8657 Japan; 7 Institute of Multidisciplinary Research for Advanced Materials, Tohoku University, 2-1-1 Katahira, Aoba-ku, Sendai, 980-8577 Japan; 8 Provost Office, Okinawa Institute of Science and Technology Graduate University (OIST), 1919-1, Tancha, Onna-son, Kunigami-gun, Okinawa, 904-0495 Japan; 9 Department of Cell Biology and Neuroscience, Juntendo University, Graduate School of Medicine, Tokyo, 113-8421 Japan; 10 Graduate School of Science, Kyoto University, Kitashirakawa Oiwake-cho, Sakyo-ku, Kyoto, 606-8502 Japan

**Keywords:** Cryoelectron microscopy, Structural biology

## Abstract

Endothelin type B receptor (ET_B_R) plays a crucial role in regulating blood pressure and humoral homeostasis, making it an important therapeutic target for related diseases. ET_B_R activation by the endogenous peptide hormones endothelin (ET)−1–3 stimulates several signaling pathways, including G_s_, G_i/o_, G_q/11_, G_12/13_, and β-arrestin. Although the conserved NPxxY motif in transmembrane helix 7 (TM7) is important during GPCR activation, ET_B_R possesses the lesser known NPxxL motif. In this study, we present the cryo-EM structure of the ET_B_R–G_i_ complex, complemented by MD simulations and functional studies. These investigations reveal an unusual movement of TM7 to the intracellular side during ET_B_R activation and the essential roles of the diverse NPxxL motif in stabilizing the active conformation of ET_B_R and organizing the assembly of the binding pocket for the α5 helix of G_i_ protein. These findings enhance our understanding of the interactions between GPCRs and G proteins, thereby advancing the development of therapeutic strategies.

## Abstract (web summary)

Tani et al. present the cryo-EM structure of the endothelin type B receptor (ETBR) in complex with the Gi protein, emphasizing the movements of transmembrane helix 7 during ETBR activation. The study underscores the crucial role of the NPxxL motif in stabilizing the receptor's active state and organizing the Gi protein binding site, enhancing our understanding of GPCR activation and aiding therapeutic development.

## Introduction

The endothelin (ET) family comprises three endogenous isoforms (ET-1–3), each of which contains 21 amino acid residues and two intramolecular disulfide bonds. ET-1, the primary isoform in the human cardiovascular system, is one of the most abundant, potent, and long-lasting constrictors of blood vessels. ET-1 plays a significant role in physiological processes, such as modulation of basal vascular tone, regulation of sodium balance, development of neural crest cells, and cell proliferation, and development of pathophysiological conditions, such as cardiovascular disease, neurological disorders, renal disease, and cancer^1–5^. The ET family exerts its effects through ET receptors, specifically subtypes ET_A_ and ET_B_ (ET_A_R and ET_B_R, respectively), which belong to the β-subfamily of class-A G-protein-coupled receptors (GPCRs). The ET-bound receptors transmit signals through heterotrimeric G proteins with promiscuous coupling properties and also interact with β-arrestins^2,6–8^.

GPCRs mediate cellular responses to various extracellular molecules, including lipids, nucleosides, neurotransmitters, hormones, and proteins. Ligand binding triggers structural changes in GPCRs, initiating signal transmission. Agonist-mediated GPCR activation is well understood, with specific conserved sequence regions, including C^6.47^W^6.48^xP^6.50^, P^5.50^I^3.40^F^6.44^, N^7.49^P^7.50^xxY^7.53^, and D^3.49^R^3.50^Y^3.51^ motifs (using Ballesteros–Weinstein numbering^9^ for class-A GPCRs), playing successive roles^10–16^. Furthermore, three highly conserved residues: R^3.50^ in DRY, Y^5.58^, and Y^7.53^ in NPxxY, play key roles in activating class-A GPCRs^11–15^. Y^7.53^ in NPxxY acts as a switch for water rearrangement, in addition to the inward movement of the cytoplasmic end of TM7 during activation^17^. During this process, N^7.49^ from NPxxY interacts directly with the conserved D^2.50^ and Y^7.53^ interacts with the highly conserved Y^5.58^ in TM5, either directly or through a bridging water molecule known as the “water lock” in the active state^18,19^. Because Y^5.58^ in TM5 undergoes rotation during activation and then stabilizes the orientation of R^3.50^ through a hydrogen bond, Y^7.53^ in NPxxY indirectly stabilizes the orientation of R^3.50^ in DRY. Thus, R^3.50^, Y^5.58^, and Y^7.53^ change their interactions during activation and structurally cooperate to generate the active state of class-A GPCRs.　However, some class-A GPCRs have unique motifs such as the NPxxL found in ET_B_R; how these conserved or divergent motifs contribute to the formation of binding pockets for heterotrimeric G proteins is unclear.

We determined the crystal structures of thermostabilized ET_B_R in three forms: ET-1-bound, ligand-free, and antagonist bosentan-bound^20–22^. Although the ET-1-bound ET_B_R structure detailed the binding of ET-1 to the receptor, it did not explain the activation mechanism, because the intracellular side was fixed in an inactive state by the insertion of T4 lysozyme into ICL3. To better understand ET_B_R activation by ET-1 and its coupling with G proteins, we report the structure of the ET-1-bound ET_B_R–G_i_ complex, determined using cryo-electron microscopy (cryo-EM), and further evaluated with MD simulations and mutagenesis studies. We identified a unique feature—the downward motion of TM7 during activation through a diverse NPxxL motif. This motion stabilized the active conformation of ET_B_R, leading to the formation of a hydrophobic binding pocket for the C-terminal α5 helix of Gα_i_.

## Results

### Overall structure of the ET-1-bound ET_B_R–G_i_ complex

**Fig. 1:**
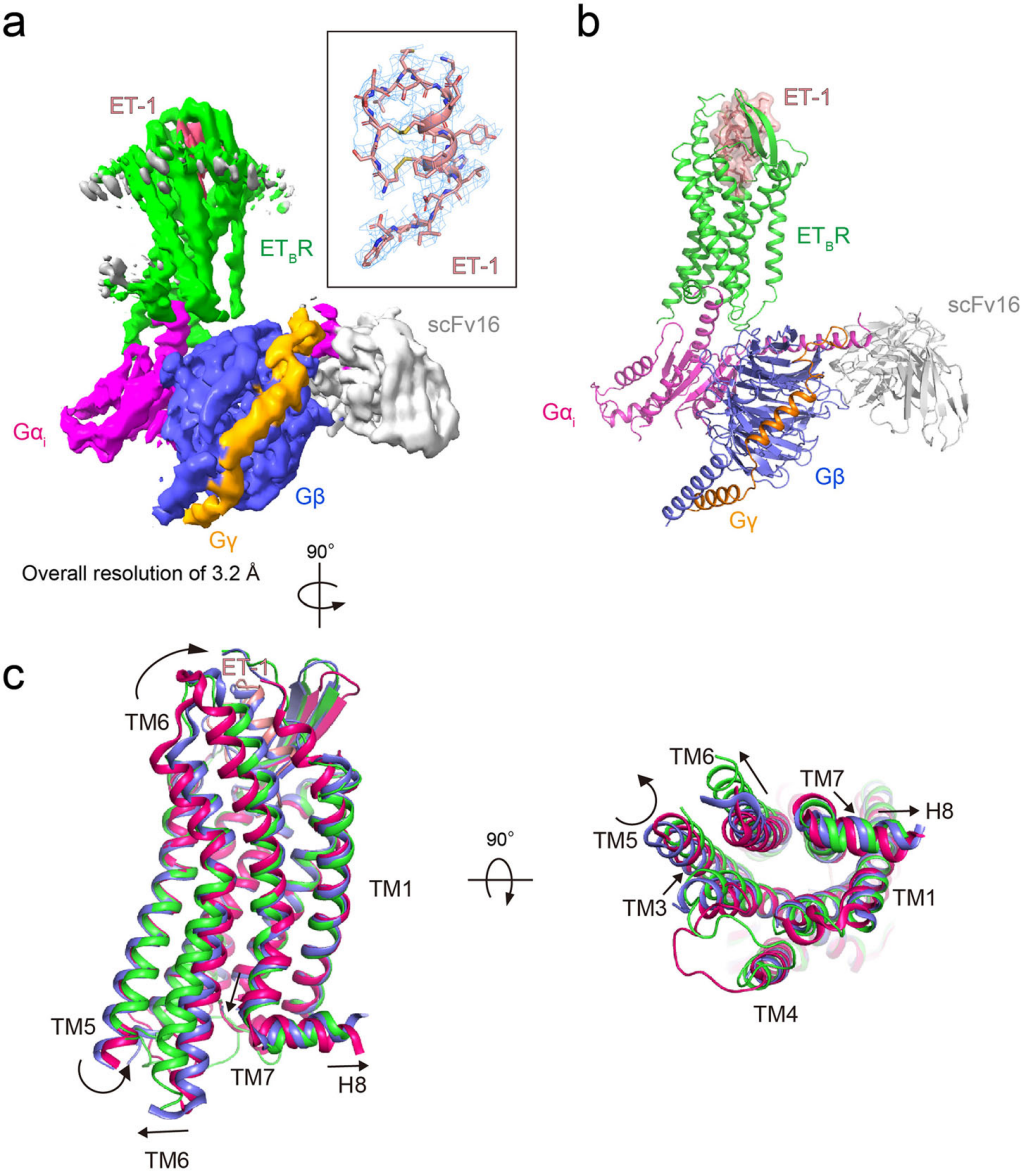
Cryo-EM structure of the ET-1–ET_B_R–DNG_i_ complex. **a** Cryo-EM density map of the ET-1–ET_B_R–DNG_i_–scFv16 complex. Green: ET_B_R, salmon: ET-1, magenta: DNGα_i_ Ras-like domain, blue: Gβ, orange: Gγ, and gray: scFv16. The inset shows the ET-1 model with the corresponding density at a contour level of 4.0 σ. **b** Molecular model of the ET-1–ET_B_R–DNG_i_ complex in the same view and color scheme as in **a**. **c** Comparison of the G_i_-stabilized active state of ET-1–ET_B_R (green), partially active state of ET-1–ET_B_R (blue), and bosentan-bound inactive ET_B_R (red). Black arrows represent helical movements from inactive to active state of ET_B_R.

To facilitate complex formation, ET_B_R and G_i1_ heterotrimer were expressed separately in Sf9 insect cells and combined after purification in lauryl maltose-neopentyl glycol (LMNG) and cholesteryl hemisuccinate (CHS). ET_B_R was stabilized by introducing the R124Y^1.55^ thermostabilizing mutation, which does not reduce ET-1 binding affinity and G-protein coupling ability^21^. Stabilization of the ET_B_R–G_i_ complex was achieved by introducing four dominant negative mutations into the Gα_i1_ subunit^23^. In addition, scFv16^24^ was used to stabilize interactions between the α_i1_ and β subunits (Supplementary Figs. 1, 2). First, the structure of the ET-1-bound ET_B_R–wild-type G_i1_–scFv16 complex was analyzed by single particle cryo-EM at a global resolution of 4.6 Å (Table 1, Supplementary Figs. 1, 3). To improve resolution, the structure of the complex, including the dominant negative Gα_i1_ subunit (DNGα_i1_), was determined with a global resolution of 3.2 Å (Fig. 1, Table 1, Supplementary Figs. 2, 4). Furthermore, we performed focused 3D refinement to obtain receptor densities at a resolution of 3.6 Å. Receptor density was assessed in the ET_B_R–DNG_i1_ complex after adjusting the alignment center to the receptor (Table 1, Supplementary Figs. 2, 5). Both ET_B_R–G_i_ complex models are nearly identical—their Cα atoms have an RMSD of 0.662 Å (Supplementary Fig. 6a). Compared with the ET-1 bound ET_B_R model in ET_B_R–DNG_i1_, the small RMSD values of the Cα atoms and the similar residue conformations in the other two models indicate they are nearly identical (0.391 Å for ET_B_R–wild-type G_i1_ and 0.364 Å for the focused 3D refinement of ET_B_R) (Supplementary Fig. 6b, c). The G_i1_-bound ET_B_R structure displayed a typical outward movement of the cytoplasmic side of TM6 to a moderate extent (approximately 7 Å), similar to other class-A G_i_-bound GPCRs (Supplementary Fig. 7). We used the ET-1-bound ET_B_R–DNG_i1_–scFv16 complex as the ET_B_R–G_i1_ complex and analyzed structural changes in detail.

**Table 1:** Cryo-EM data collection, refinement and validation statistics of the ET_B_ receptor-G_i_ complexes

	ET-1 bound ET_B_R-DNG_i_-scFv16 (EMDB-38740, PDB-8XWP)		ET-1 bound ET_B_R-DNG_i_-scFv16 (focused ET_B_R) (EMDB-60404, PDB-8ZRT)	ET-1 bound ET_B_R-wild type G_i_-scFv16 (EMDB-38741, PDB-8XWQ)
Data collection and processing				
Microscope	JEOL CRYO-ARM300			TF Talos Arctica
Camera	K3			Falcon III
Magnification	60,000	100,000		92,000
Voltage (kV)	300	300		200
Electron exposure (e–/Å^2^)	53.3	49.2		40
Defocus range (μm)	–0.5 to –2.7	–0.6 to –3.5		–0.7 to –2.9
Calibrated pixel size (Å)	0.816	0.507		1.094
Detector physical pixel size (μm)	5			14
Symmetry imposed	C1			C1
Initial particle images (no.)	1,193,302	1,757,339	1,038,215	954,972
Final particle images (no.)	556,576	481,639	401,671	278,209
Map resolution (Å)	3.2		3.6	4.6
FSC threshold	0.143		0.143	0.143
Map resolution range (Å)	5.4–3.1		8.7–3.6	6.9–4.4
Refinement				
Initial model used (PDB code)	5GLH, 6OS9		8XWP	5GLH, 6OS9
Model resolution (Å)	3.2		4.1	4.6
FSC threshold	0.5		0.5	0.5
Model resolution range (Å)	120–3.2		120–3.6	126–4.6
Map sharpening *B* factor (Å^2^)	–100		-100	–263
Model composition				
Non-hydrogen atoms	9076		2322	9076
Protein residues	1163		303	1163
*B* factors (Å^2^)				
Protein	80.7		48.9	191.8
R.m.s. deviations				
Bond lengths (Å)	0.002		0.003	0.003
Bond angles (°)	0.473		0.693	0.546
Validation				
MolProbity score	2.31		1.82	2.59
Clashscore	8.97		13.56	14.24
Poor rotamers (%)	5.56		0.81	7.98
Ramachandran plot				
Favored (%)	96.06		96.93	96.33
Allowed (%)	3.94		3.07	3.67
Disallowed (%)	0.00		0.00	0.00

### Structure of G_i_-stabilized active ET_B_R

**Fig. 2:**
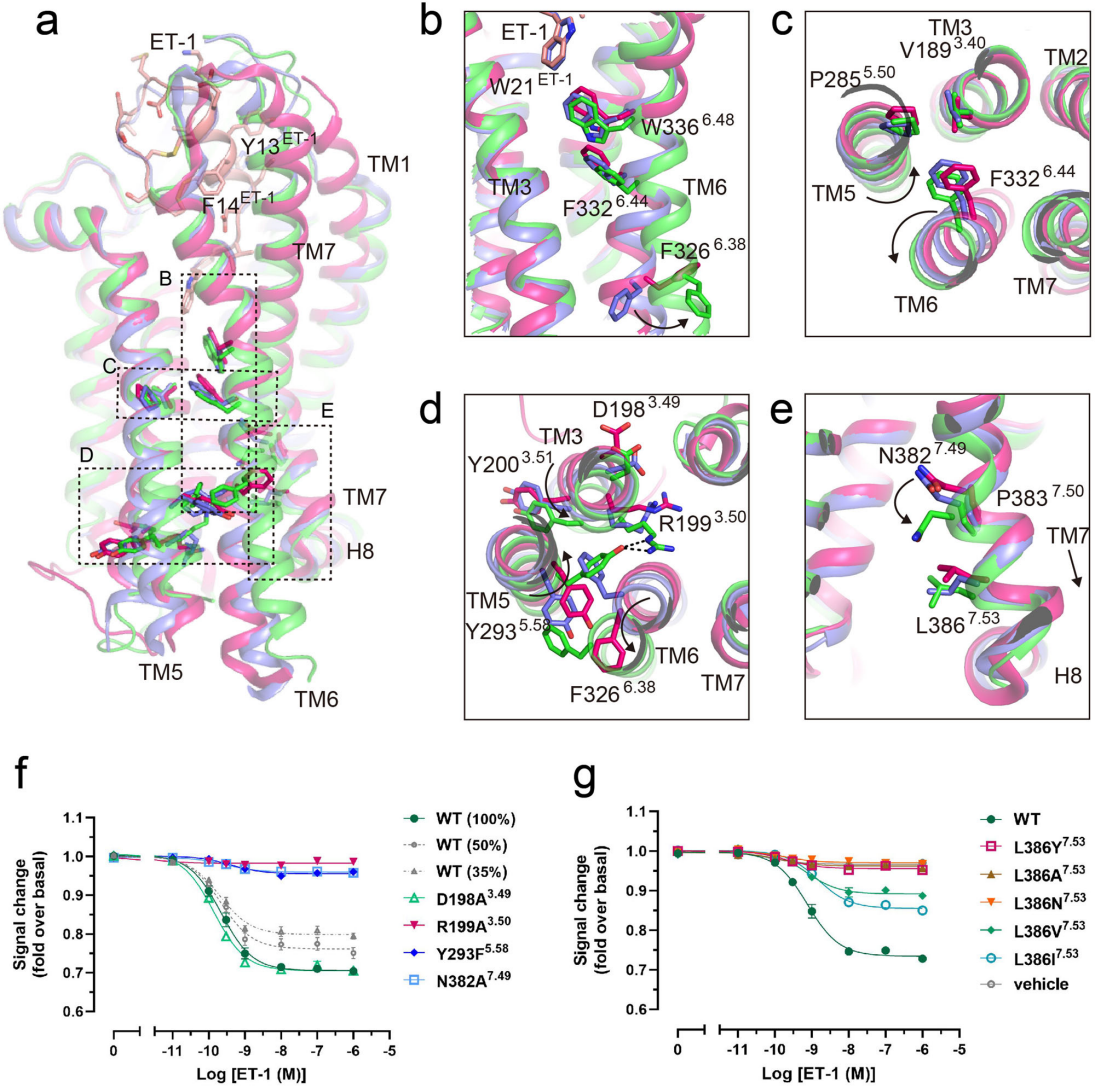
G_i_-coupled ET_B_R is in an active conformation. **a** Superposition of the G_i_-bound ET_B_R structure (green) with the partially active-state crystal structure of ET-1-bound ET_B_R (blue) and the inactive-state crystal structure of the antagonist bosentan-bound ET_B_R (magenta). **b**–**e** Close-up views of conserved motifs involved in receptor activation. Arrows indicate the repositioning of side chains from the inactive to active state. **f**, **g** Concentration–response curves for ET-1-induced G_i_ signaling activity in the NanoBiT G-protein dissociation assay of ET_B_R–wild-type (WT) and mutant receptors. Symbols and error bars represent mean and standard error of the mean (SEM), respectively, from three independent experiments, each performed in duplicate or triplicate. Signaling of reduced amounts of WT ET_B_R (% of plasmid DNA transfected) for G_i_ is shown in gray. Data for these figures and expression levels of WT and mutant receptors measured by [^125^I]ET-1 binding are shown in Supplementary Fig. 8 and Table 1a, b.

The mode of ET-1 binding in the ET_B_R–G_i_ complex closely resembled that of the crystal structure of ET-1-bound ET_B_R. Y13^ET-1^ and F14^ET-1^ in the helical region of ET-1 played a pivotal role in the compact assembly of the N-terminal tail and the extracellular side of TM7, initiating helical rearrangements of ET_B_R (Fig. 2a). This assembly is essential for full G-protein activation^25^. The C-terminal region of ET-1 (L17^ET-1^–W21^ET-1^) fits into the transmembrane orthosteric pocket of the receptor, interacting with many hydrophobic (I157^2.60^, L277^5.42^, L339^6.51^, etc.) and hydrophilic (including K182^3.33^, K273^5.38^, R343^6.55^, D368^7.35^, etc.) residues^20^. The C-terminal side chain of W21^ET-1^ directly interacted with W336^6.48^ in the CWxP motif (Fig. 2b). Interactions between ET-1 and ET_B_R, both in the transmembrane region surrounding the C-terminal region of ET-1 and close to the extracellular side, played a role in ET_B_R activation. These ligand–receptor interactions influenced the helical rearrangement of ET_B_R through the conserved V189^3.40^P285^5.50^F332^6.44^ motif, resulting in an inward rotation of R199^3.50^ and Y293^5.58^, an outward movement of the cytoplasmic side of TM6 (Fig. 2b, c), and crevice formation on the cytoplasmic side of the receptor to accommodate Gα_i_.

**Fig. 3:**
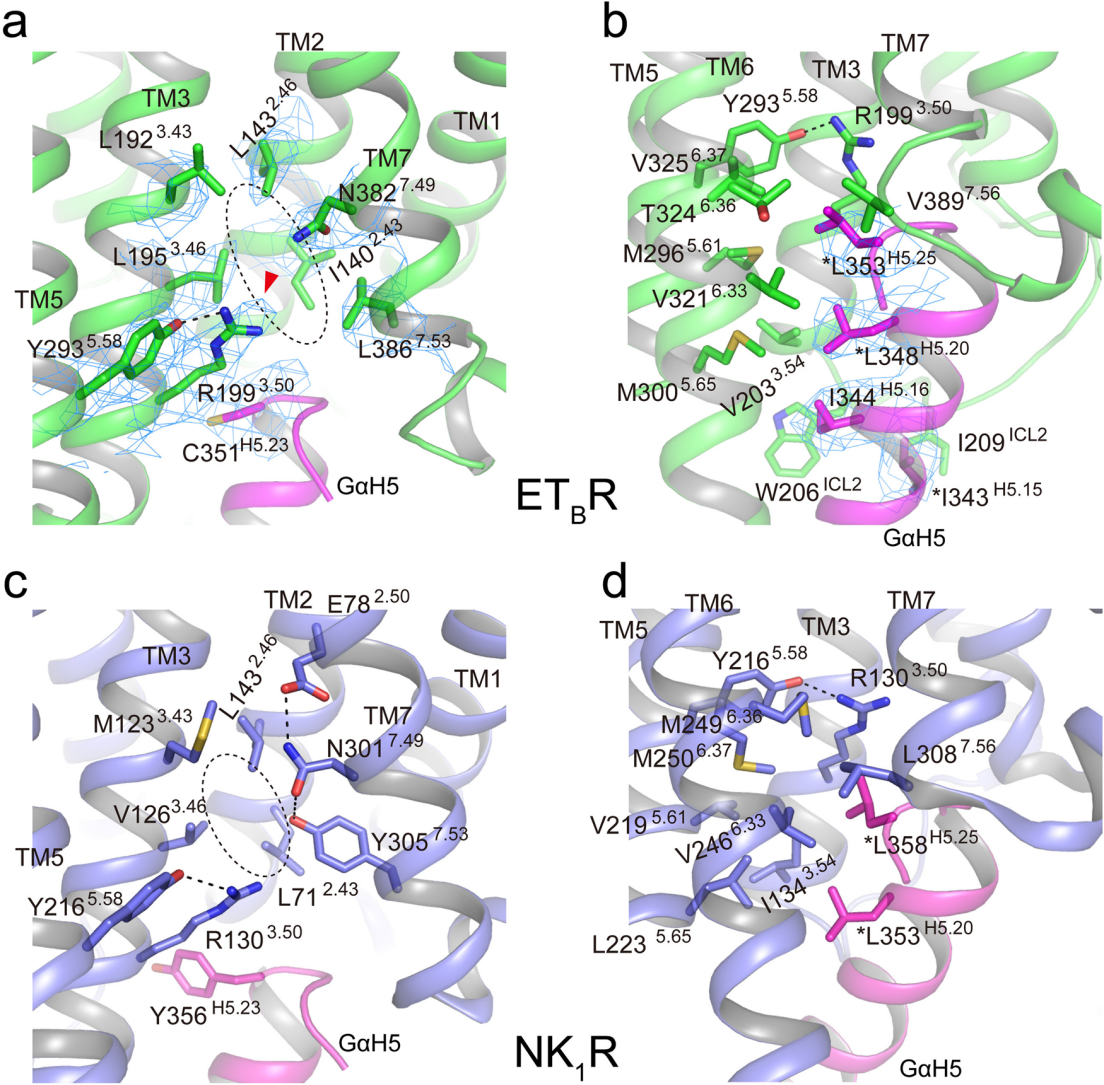
Hydrophobic interactions between ET_B_R and NK_1_R in the active state. Hydrophobic interactions around R^3.50^ and L/Y^7.53^ of ET_B_R (**a**, **b**) and NK_1_R (**c**, **d**), respectively. **a** The downward motion of TM7 of ET_B_R is stabilized by N382^7.49^ and L386^7.53^ in the NPxxL motif through a series of hydrophobic interactions with I140^2.43^, L195^3.46^, etc. The density around all rendered residues at a contour level of 5.0 σ is shown as a mesh. **b** The large hydrophobic side chains of L348^H5.20^ and L353^H5.25^ of Gα_i_ penetrate deeply into the hydrophobic pocket formed by TM3, TM5, TM6, and TM7 of ET_B_R. I343^H5.15^ and I344^H5.16^ form additional interactions with ICL2. The density around the rendered residues of the α5 helix of Gα_i_ is shown as a mesh at a contour level of 5.0 σ. **c** The downward motion of TM7 of NK_1_R is stabilized by E78^2.50^, N301^7.49^, and Y305^7.53^ in NPxxY through a series of hydrogen-bond interactions as well as hydrophobic interactions with L71^2.43^, V126^3.46^, etc. **d** The large hydrophobic side chains of L353^H5.20^ and L358^H5.25^ of Gα_q_ penetrate deeply into the hydrophobic pocket formed by TM3, TM5, TM6, and TM7 of NK_1_R. Identical residues among G_i_, G_o_, and G_s_ are denoted by “*” before the amino acid label, but a conserved residue (L348^H5.15^ of Gα_q_) in **d** was omitted because it does not contact the receptor. The NPxxL motif leads to the formation of a larger cavity than NPxxY (indicated by a dashed oval).

As observed in other class-A GPCRs, the outward shift of TM6 disrupted the salt bridge between D198^3.49^ and R199^3.50^ of DRY, seen in the ET-1-bound inactive ET_B_R. R199^3.50^ extended toward TM7, stabilized by Y293^5.58^ through hydrogen bonding (Fig. 2d, Supplementary Fig. 8a). A simultaneous downward displacement (~ 1.5 Å) at the NPxxL motif (N382^7.49^, P383^7.50^, and L386^7.53^ in ET_B_R instead of Y^7.53^) was observed in the ET-1–ET_B_R–G_i_ complex (Fig. 2e). N382^7.49^ extended toward R199^3.50^, and L386^7.53^ formed a hydrophobic interaction with I140^2.43^ to stabilize the helical contacts between TM2 and TM7 (Figs. 2e and 3a). Because the residue at 386^7.53^ was leucine, and not the conserved tyrosine, the hallmark water-mediated hydrogen bonding network, including Y^7.53^ and R^3.50^, which is characteristic of class-A GPCR activation (Supplementary Fig. 8b), was not formed.

Structural comparisons across class-A GPCRs indicated a conserved rearrangement of residue contacts at positions 3.46 and 7.53 upon activation^10^. In many class-A GPCRs with the conserved Y^7.53^ sequence in the active state, distances between residues 3.46 and 7.53 are typically ≤4.5 Å, allowing for hydrophobic or van der Waals interactions. However, for ET_B_R, the distance between L195^3.46^ and L386^7.53^ was approximately 7.3 Å without direct contact, because the absence of Y^7.53^ and downward shift of TM7 created a space between them (Fig. 3a). The side chains of the rotated R199^3.50^ and N382^7.49^ extended toward this space, where possible water molecules were detected (Fig. 3a, Supplementary Fig. 8a). In a later section, we validated the presence of water molecules using MD simulations.

The downward shift of TM7 was stabilized by a hydrophobic interaction between L386^7.53^ and I140^2.43^, which simultaneously interacted with L195^3.46^ (Fig. 3a). Despite the considerable distances between residues 3.46 and 7.53, precluding direct contacts, this conformation could be maintained. Furthermore, V389^7.56^, located one turn downward from L386^7.53^ in TM7 of ET_B_R, contacted T324^6.36^ in TM6, as seen in other class-A GPCRs with the conserved Y^7.53^ (Fig. 3b). Remarkably, residues S390^8.47^ and V325^6.37^, which are adjacent and play crucial roles as binding sites for the α5 helix of Gα_i_ (described in the next section), were appropriately arranged in the active conformation of ET_B_R through the downward motion of TM7. Hence, although the unique 382N^7.49^Pxx386L^7.53^ motif creates an unusual space between L195^3.46^, R199^3.50^, N382^7.49^, and L386^7.53^ (Fig. 3a, Supplementary Fig. 8a), a binding pocket for the α5 helix of Gα_i_ was established in the active structure of ET_B_R (described ahead). This unusual space can be observed in the area between V126^3.46^ and Y305^7.53^ of NK_1_R, comprising the NPxxY motif^26,27^. The surrounding area demonstrates an active conformation like ET_B_R, characterized by a downward shift of the cytoplasmic end of TM7, contrasting with the other GPCRs with the NPxxY motif (Fig. 3c, Supplementary Fig. 8; see the Discussion section).

The biological importance of these interactions in the active conformation of ET_B_R was confirmed through the dissociation of heterotrimeric G proteins associated with its activation^7,28^ (Fig. 2f, g, Supplementary Fig. 9, Table 1). Mutations R199^3.50^A, Y293^5.58^F, and N382^7.49^A, resulted in nearly complete impairment in the G_i_-protein dissociation assay. Additionally, although hydrophobic mutations of L386^7.53^ to Ile and Val reduced dissociation activities by approximately 50% when considering their expression levels (Supplementary Table 1), mutations of L386^7.53^ to hydrophilic or small residues, such as Tyr, Ala, or Asn, resulted in severely impaired activities. The importance of these residues in forming the active conformation was confirmed through the GloSensor cAMP accumulation assay (Promega) through G_s_ coupling. We observed severe impairment due to mutations, which is consistent with the findings of the G_i_ dissociation assay (Supplementary Fig. 10a, b, Supplementary Table 2). Thus, interactions between R199^3.50^, Y293^5.58^, and N382^7.49^ are biologically essential for G_i_-protein activation, and the bulky hydrophobic residue leucine at 386^7.53^ is important for the active conformation of ET_B_R.

### ET_B_R–G_i_ interface

The structure of the ET_B_R–G_i_ complex (Fig. 1a, Supplementary Fig. 7) revealed a mode of interaction similar to that in other G_i_-bound receptors. However, the interactions between ET_B_R and G_i_ were exclusively mediated through the α5 helix of Gα_i_. This helix binds ET_B_R in a more vertical orientation in ET_B_R–G_i_ than in other GPCR–G_s_ or G_q_ structures (Supplementary Fig. 7). Consequently, the C-terminus of the α5 helix of Gα_i_ dominantly bound ET_B_R, which confined the ET_B_R–G_i_ interface within a relatively narrow area.

**Fig. 4:**
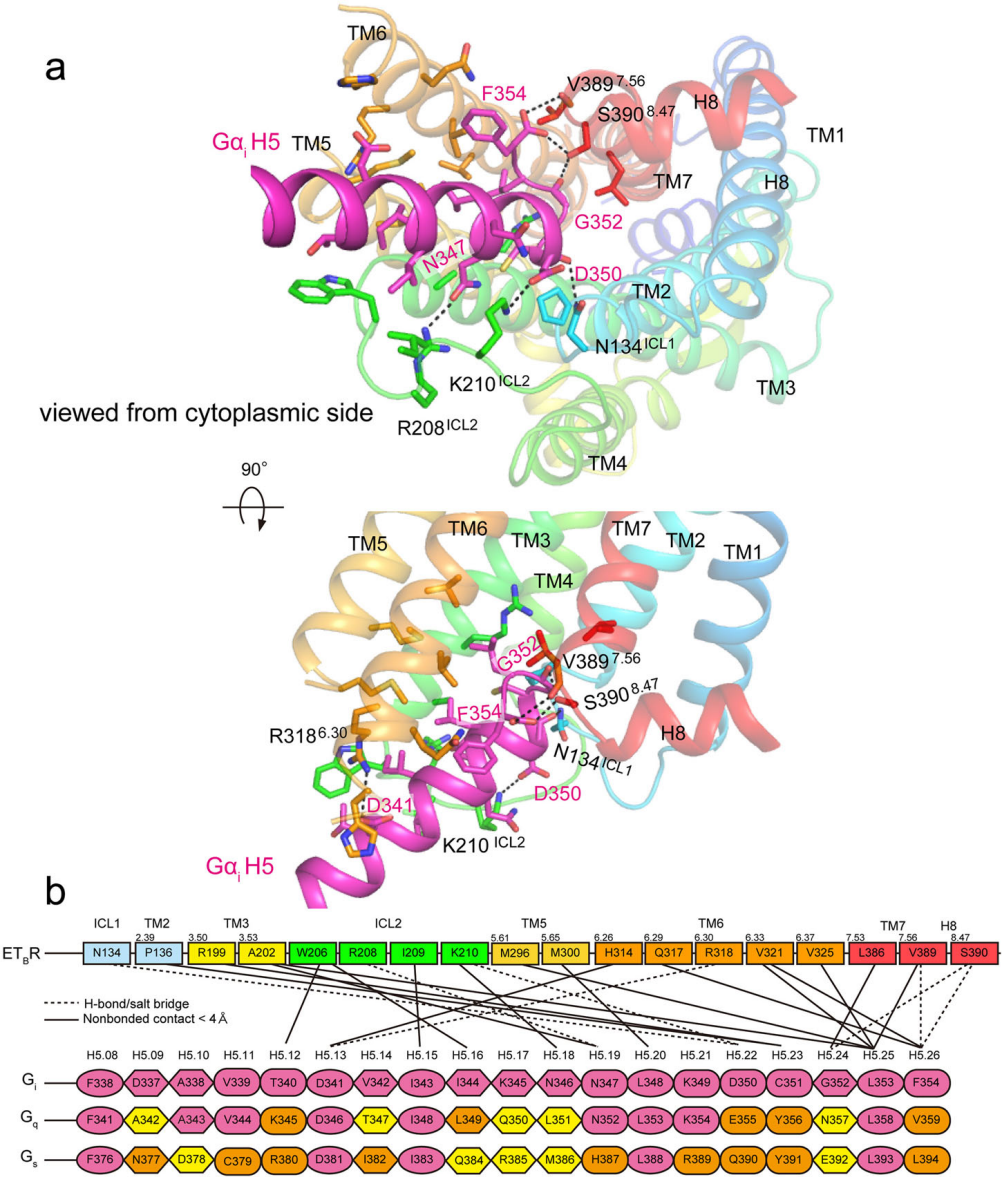
Interface between ET_B_R and Gα_i_. **a** Close-up view of the interaction between ET_B_R and the α5 helix of Gα_i_. Hydrogen bonds are indicated by black dotted lines. **b** Schematic representation of direct contacts between ET_B_R and the α5 helix of Gα_i_. Hydrogen-bonded and hydrophobic contacts are indicated by dashed and solid lines, respectively. Receptor residues involved in hydrogen bonding are numbered according to Ballesteros–Weinstein numbering^9^, and Gα_i_ residues involved in hydrogen bonding are numbered according to CGN numbering^29^. Gα_i_ and conserved Gα_q_ and Gα_s_ residues are in magenta, homologous residues of Gα_q_ and Gα_s_ are in orange, and others are shown in yellow.

A significant interface between ET_B_R and Gα_i1_ was formed by TM3, TM5, TM6, TM7, ICL1, and ICL2 of the receptor, in addition to the last 15 residues of the C-terminal α5 helix (residues 340–351) and the following three-residue wavy hook (352-GLF-354) of Gα_i_ (Fig. 4a). In detail, as observed in many G_i_-bound GPCR complexes, the apex of the α5-helix engaged with the end of TM7 and helix 8. At this interface, the backbone carbonyl of G353^H5.24^ (superscripts refer to the CGN numbering system)^29^ and C-terminal carboxylate of F354^H5.26^ formed hydrogen bonds with the side chain of S390^8.47^ and the backbone carbonyl of V389^7.56^ of ET_B_R. Gα_i_ residues, including D341^H5.13^, N347^H5.19^, and D350^H5.22^, established four hydrogen bonds with ET_B_R residues N134A^ICL1^, R208^ICL2^, K210 ^ICL2^, and R318^6.30^ (Supplementary Table 3).

However, the amino acid residues located between the cytoplasmic cleft of ET_B_R and the α5 helix of Gα_i_ predominantly formed van der Waals interactions for the pairs C351^H5.23^–R199^3.50^ and N347^H5.19^–A202^3.53^ (Fig. 4, Supplementary Table 3). Notably, the large side chains of L348^H5.20^ and L353^H5.25^ nestled deeply into the hydrophobic pocket formed by V203^3.54^, M296^5.61^, M300^5.65^, V321^6.33^, V325^6.37^, and V389^7.56^ in TM3, TM5, TM6, and TM7 of ET_B_R (Fig. 3b). The hydrophobic residues I344^H5.16^ and I343^H5.15^ formed interactions with W206^ICL2^ and I209^ICL2^, respectively. Although residues at the C-terminal side of the α5 helix of Gα_i_ interacted with residues within the ET_B_R hydrophobic pocket, residues in the middle part of the α5 helix, such as T340^H5.12^, D341 ^H5.13^, and I343 ^H5.15^, interacted with residues in ICL2, such as W206^ICL2^ and I209^ICL2^, or close to ICL3, such as H314^6.26^ and R318^6.30^. Thus, the α5 helix of Gα_i_ binding to ET_B_R showed a relatively vertical orientation (Supplementary Fig. 7). This resulted in a shorter cytoplasmic side of TM5 compared with other class-A GPCRs, and the ICL2 of ET_B_R formed a flexible loop.

### ET_B_R-G_i_ dissociation assay

**Fig. 5:**
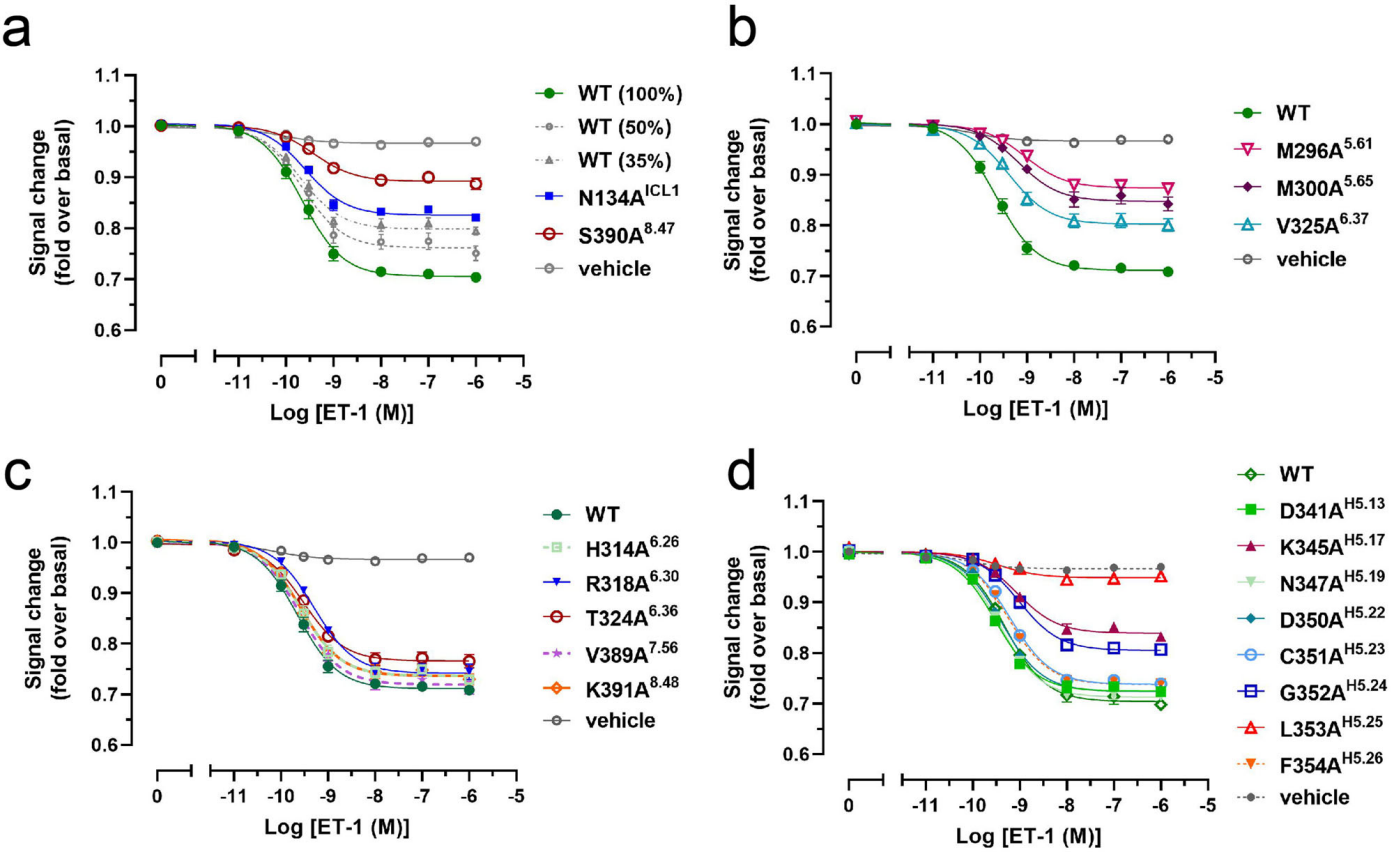
Validation of the interface residues of the ET_B_R–G_i_ complex in the NanoBiT G_i_-protein dissociation assay. Symbols and error bars represent mean and standard error of the mean (SEM), respectively, from three independent experiments, each performed in duplicate or triplicate. **a**–**c** The replaced interface residues of ET_B_R were examined. Data for these figures and the expression levels of WT and mutant receptors are shown in Supplementary Table 1c–e. **d** The replaced interface residues of Gα_i_ were examined. Mutant G_i_ show luminescence counts comparable with those of WT. Data for this figure are shown in Supplementary Table 1f.

These structural observations were validated using an ET_B_R-stimulated G_i_-protein dissociation assay to examine the recognition determinants. The each ET_B_R mutant receptor retained the affinity for ET-1 comparable to that of the wild-type (Supplementary Table 4). Among ET_B_R mutations, S390^8.47^A, M296^5.61^A, M300^5.65^A, and V325^6.37^A substantially reduced the coupling between the receptor and Gα_i_ by approximately 50%, whereas N134^ICL1^A, H314^6.26^A, R318^6.30^A, V389^7.56^A, and K391^8.48^A mutations retained comparable or slightly reduced activities compared with wild-type, considering the expression of mutant receptors (Fig. 5a–c, Supplementary Table 1c–e). By contrast, among Gα_i_ mutations, replacing L353^H5.25^ with alanine severely impaired coupling with ET_B_R, whereas G352A^H5.24^ and K345A^H5.17^ mutations decreased coupling by 50%. C351A^H5.23^ and F354A^H5.26^ mutations showed a slight reduction, whereas D341A^H5.13^ and D350A^H5.22^ mutations did not exhibit marked defects (Fig. 5d, Supplementary Table 1f). These findings are consistent with extensive mutagenesis studies of Gα_i1_ on the stability and formation of the rhodopsin–G_i_ complex, where L353A^H5.25^, G352A^H5.24^, and L348A^H5.20^ substitutions severely impaired coupling, and C351A^H5.23^, K345A^H5.17^, and I344A^H5.16^ substitutions reduced complex formation efficiencies to approximately 60%^30^. Therefore, coupling efficacies affected by mutations in ET_B_R and the α5 helix of Gα_i_ corresponded well with each other, reflecting their interactions at the observed interface of the complex. Notably, interactions at the end of TM7 and helix 8 of ET_B_R with the C-terminus of the Gα_i_ α5 helix, as well as the hydrophobic pocket composed of V203^3.54^, M296^5.61^, M300^5.65^, and V325^6.37^ with the C-terminal L348^H5.20^ and L353^H5.25^ of Gα_i_ α5 helix, play crucial roles in ET_B_R–G_i_ coupling.

Most residues of the C-terminal α5 helix (T340–F354) interacted with ET_B_R in the complex, except K345^H5.17^, which interacted with F354^H5.26^ through a cation–π interaction, and with D341^H5.13^ and E318^h4s6.12^ through salt bridges within Gα_i1_ (Supplementary Fig. 11). In the GDP-bound form, K345^H5.17^ did not interact with D341^H5.13^ or E318^h4s6.12^, which was originally located at the end of the β6 sheet. The translation and twist of the α5 helix during coupling with ET_B_R led to K345^H5.17^ interacting with D341^H5.13^ and E318^h4s6.12^. This interaction stabilized the twisted α5 helix and the conformation of the shortened β6 sheet as well as the GDP-released β6-α5 loop. The K345A^H5.17^ mutation led to an approximately 50% reduction in the G_i_ dissociation assay (Fig. 5d) and the rhodopsin–G_i_ complex formation assay^30^, indicating that K345^H5.17^ plays a fundamental role in G_i_ activation. This role includes modulating the location of C-terminal F354^H5.26^ and stabilizing the ET_B_R–G_i_ complex.

### ET_B_R coupled through the C-terminus of Gα

The α5 helix comprises conserved and variable residues across Gα proteins and could serve as a common mode of interaction with various types of GPCRs or as a selective mode of interaction based on receptor specificity^29^. The structural insights provided by the ET_B_R–G_i_ structure, in addition to the results of biological validation, suggest that conserved hydrophobic residues, particularly L348^H5.20^ and L353^H5.25^, play pivotal roles in coupling (Figs. 4b and 5d). These residues form numerous contacts with specific residues in the hydrophobic binding pocket of ET_B_R. When these residues are substituted with others, coupling is significantly impaired (Fig. 5b, Supplementary Fig. 10c). Additionally, subtype-specific residues involved in Gα selectivity, such as C351^H5.23^ and G352^H5.24^, occupied crucial positions in the complex and established contacts with the central residues of ET_B_R, including R199^3.50^ and L386^7.53^ (Figs. 2f, g, 5a, d, Supplementary Fig. 10, Tables 1, 2). Notably, the primary interactions of ET_B_R with the α5 helix of Gα_i_ are limited to the transmembrane area. This is because the binding of the C-terminal α5 helix to ET_B_R occurs in a relatively vertical orientation, and ICL2 of ET_B_R is a flexible loop. Consequently, in the coupling of ET_B_R with other subfamilies, such as G_s_, G_q_, and G_12_, it is likely that the conserved L348^H5.20^ and L353^H5.25^ continue to play central roles as binding partners through a common mode of interactions (Fig. 3b, d). ET_B_R may further adapt to selectively accommodate subtype-specific residues, such as H5.23 and H5.24, based on the requirements of the G-protein subfamily^29^. These distinctive features would enable ET_B_R to exhibit promiscuity in coupling with G-protein subfamilies^2,6,7^.

### ET_B_R–G_i_ interactions in molecular dynamics simulations

**Fig. 6:**
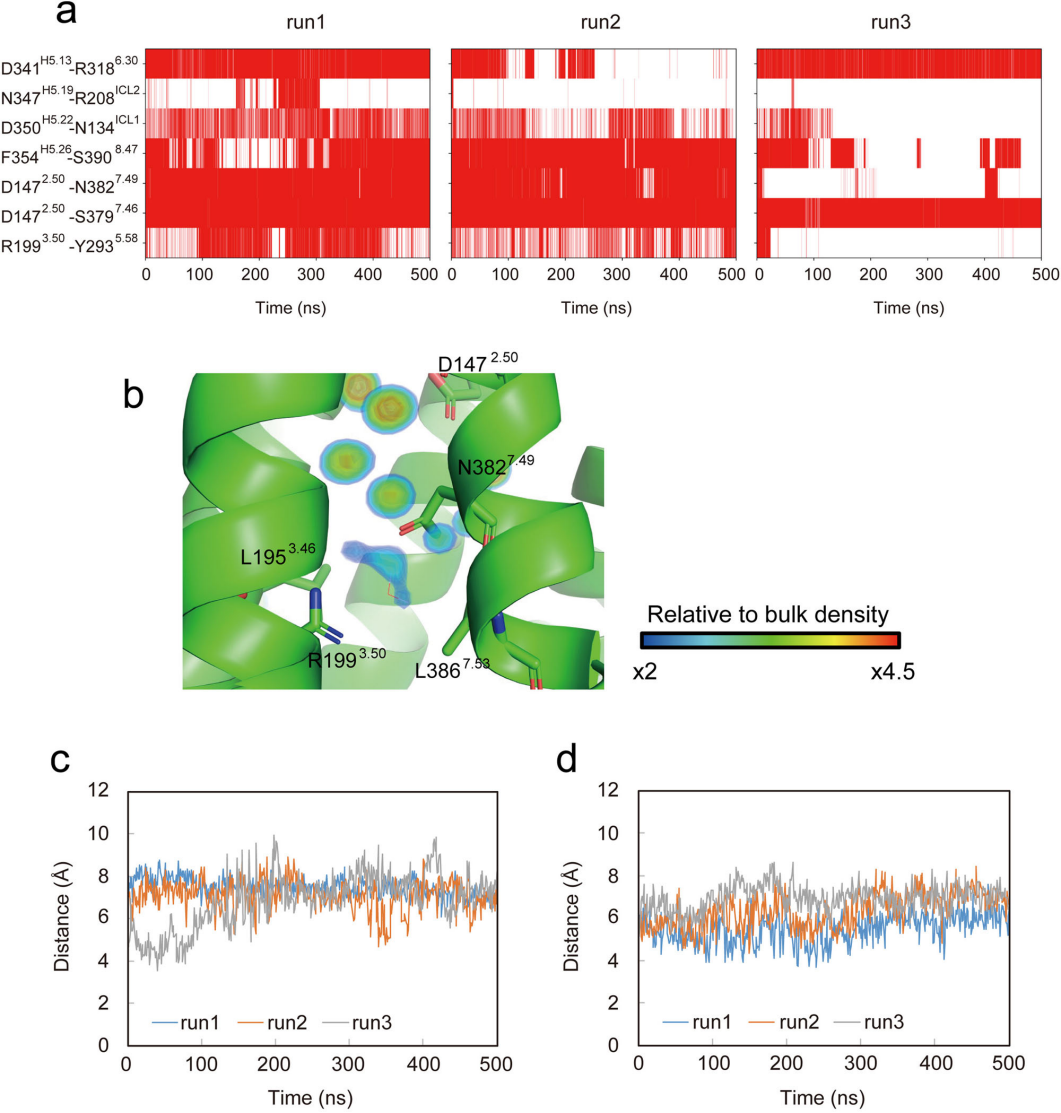
Intermolecular and intramolecular interactions observed in MD simulations. **a** Hydrogen-bond interactions in each run are represented by red lines. **b** Water densities in the cavity formed by transmembrane regions TM3, TM6, and TM7 in run 1 are superposed on the initial structure. Time evolution of distances between R199^3.50^ and N382^7.49^ (**c**) and between L195^3.46^ and L386^7.53^ (**d**) are shown. Distances were calculated as the minimum distance between all possible pairs of heavy atoms of two residues.

We performed molecular dynamics (MD) simulations of the ET-1–ET_B_R–G_i_ complex to evaluate the key interactions for ET_B_R–G_i_ activation. The simulations, each lasting 500 ns, were repeated three times with different initial velocities. The time evolutions of the Cα RMSDs of ET_B_R, Gα_i_, Gβ, and Gγ from the initial structures are shown in Supplementary Fig. 12a. The structures of ET_B_R, Gβ, and Gγ remained stable during MD simulations with consistent RMSD values of <3 Å. However, Gα_i_ underwent substantial conformational changes due to the large flexibility of its activated form. The Cα RMSDs of ET-1 and the C-terminal α5 helix of Gα_i_ (residues 335–354) were calculated after superposing the Cα atoms of ET_B_R on those of the initial structure (Supplementary Fig. 12b). No significant change occurred in either run, indicating stable binding of ET-1 and Gα_i_ to ET_B_R. We calculated the probabilities of hydrogen-bond formation for pairs D341^H5.13^–R318^6.30^, N347^H5.19^–R208^ICL2^, D350^H5.22^–N134^ICL1^, and F354^H5.26^–S390^8.47^ to analyze the stability of intermolecular interactions (Fig. 6a, Supplementary Table 5). Hydrogen bonds for pairs D341^H5.13^–R318^6.30^ and F354^H5.26^–S390^8.47^ were stably formed with probabilities of approximately 0.7. Although the hydrogen bond between D350^H5.22^ and N134^ICL1^ was broken after 130 ns of run 3, it was formed in runs 1 and 2 with probabilities of approximately 0.7 and 0.4, respectively, indicating the formation of a weak bond. By contrast, N347^H5.19^ and R208^ICL2^ rarely formed a hydrogen bond, because R208^ICL2^ exhibited high structural flexibility. Next, we analyzed intermolecular hydrogen bonds within ET_B_R for pairs D147^2.50^–N382^7.49^, D147^2.50^–S379^7.46^, and R199^3.50^–Y293^5.58^. Hydrogen bond D147^2.50^–S379^7.46^ was stable in all runs. Hydrogen bonds for pairs D147^2.50^–N382^7.49^ and R199^3.50^–Y293^5.58^ were weak because they formed only in runs 1 and 2. Additionally, we calculated the average water occupancy in the intracellular cavity of ET_B_R using the 500 ns trajectory of run 1 to analyze water-mediated interactions (Fig. 6b). Water densities exceeding 2-fold bulk density were observed in the cavity surrounded by L195^3.46^, R199^3.50^, N382^7.49^, and L386^7.53^. Minimum distances for the pairs R199^3.50^–N382^7.49^ and L195^3.46^–L386^7.53^ settled at approximately 7 and 6 Å, respectively (Fig. 6c, d). Thus, MD simulations revealed a water-mediated hydrogen-bond network connecting the area of Y293^5.58^–R199^3.50^–water molecules–N382^7.49^–S379^7.46^–D147^2.50^ residing at the center of ET_B_R. Accordingly, a relatively bulky density at the tip of R199^3.50^ observed in the cryo-EM map can be attributed to water, contributing to the network (an arrowhead in Fig. 3a). This network was sealed by hydrophobic interactions through L195^3.46^, I140^2.43^, and L386^7.53^, and ultimately completed by the binding of the α5 helix of Gα_i_ to the receptor.

## Discussion

The diversity in residue L386^7.53^ within NPxxL in TM7 is crucial for the active conformation of ET_B_R. Surprisingly, L386Y, as well as L386N/A, mutant receptors severely impaired G-protein activation (Fig. 2g, Supplementary Fig. 10b). Only the hydrophobic mutant receptor L386I/V retained approximately 50% of the activity. The mutant receptors indicate that a bulky hydrophobic residue at position 7.53 is indispensable for the active conformation of ET_B_R. In the common rearrangement that occurs upon activation, direct contacts occur between residues at positions 7.53 and 3.46^10^. However, L386^7.53^ was distant from L195^3.46^ in ET_B_R and linked with it through I140^2.43^ through hydrophobic interactions, presumably to maintain hydrophilic interactions and form stable contacts in the active conformation of ET_B_R (Fig. 3a). In addition, downward-shifted L386^7.53^ positions V389^7.56^ one turn below in TM7 adequately to create the binding site for Gα_i_. Both V389^7.56^ and the adjacent S390^8.47^, located at the transition of TM7 to helix 8, interact with the C-terminal region of Gα_i_, specifically the backbone carbonyl of G352^H5.24^ and the C-terminal carboxylate of F354^H5.26^ (Figs. 3b and 4a). These interactions play crucial roles in coupling (Fig. 5a, d, Supplementary Fig. 10). V389^7.56^ interacts closely with T324^6.36^ in TM6, adjacent to V325^6.37^, which interacts with M296^5.61^ in TM5, under which M300^5.65^ is positioned one turn below, and which in turn is close to V203^3.54^. Altogether, V325^6.37^, M296^5.61^, M300^5.65^, and V203^3.54^ align to form a hydrophobic core to bind the C-terminal L353^H5.25^ and L348^H5.20^ of α5 helix of Gα_i_. These interactions constitute one of the primary binding determinants (Fig. 5b, d, Supplementary Fig. 10c). Coordinating with V389^7.56^, the diverse N^7.49^PxxL^7.53^ motif plays a structural role in the active conformation of ET_B_R through a downward shift, similar to NPxxY. In class-A GPCRs, approximately 4% of the receptors possess the N^7.49^P^7.50^xxX^7.53^ sequence (X is leucine, phenylalanine, threonine, histidine, and so on (GPCRdb, http://www.gpcrdb.org) on the cytoplasmic side of TM7, such as ET_A_R^31^ and GRPR/BB_2_^32^. In these receptors, L^7.53^ may contribute to the organization of a binding pocket for Gα, similar to that observed in ET_B_R. Alternatively, NK_1_R (with NPxxY) shows an unusual downward shift of the cytoplasmic end of TM7 upon activation. Because N301^7.49^ of NK_1_R forms direct hydrogen bonds with E78^2.50^ and Y305^7.53^ in the active state, and the cytoplasmic side of TM7 does not shift inward, but to the intracellular side upon activation, due to the longer side chain of E78^2.50^ at position D^2.50^ (Fig. 3c)^26,27^. Consequently, the downward-shifted L308^7.56^ one turn below Y305^7.53^ plays an essential role as a structural pivot in the active conformation, as well as a member of the hydrophobic binding site for L353^H5.20^ and L358^H5.25^ of the C-terminal α5 helix of Gα_q_, in addition to M249^6.36^, V246^6.33^, L223^5.65^, I134^3.54^, and R130^3.50^ (Fig. 3d).

Ji et al. reported cryo-EM models of ET-1-bound ET_A_R and ET_B_R coupled to miniG_s/q_, as well as a selective peptide IRL1620-bound ET_B_R coupled to G_i_, providing valuable structural insights into these complexes^31^. Their findings suggest that interface regions between ETRs and G_i/q_ in the structures of ET_A_R and ET_B_R bound to ET-1 resemble the interface observed in our ET_B_R–G_i_ complex structure. This implies that both ET-1-bound ET_A_R and ET_B_R engage G_i_ and G_q_ in a manner similar to the hydrophobic binding pocket of L348^H5.20^, L353^H5.25^, and S373/S390^8.47^, interacting with the C-terminal end of the α5 helix. However, the deposited structures (PDB code 8HCQ, 8HCX, 8HBD) show some ambiguities. Discrepancies in the extracellular region, such as lack of disulfide bonds C158/C239 and C69/C341 in ET_A_R, C174/C255 in ET_B_R, and C3/C11 in ET-1–ET_B_R, could affect structural interpretation. Furthermore, Sano et al. presented the cryo-EM structure of the ET-1–ET_B_–G_i_ complex^33^. They observed a downward shift of the cytoplasmic side of TM7, consistent with our results. Although they used different constructs for G_i_ protein, including the linker between ET_B_R and β subunit of G_i_, their findings were consistent with the overall structure of the ET-1–ET_B_R–DNG_i1_–scFv16 complex. Notably, they described binding of the C-terminal α5 helix of Gα_i_ to ET_B_R as “shallow;” however, we have highlighted that the C-terminal wavy hook of Gα_i_ is in a relatively deeper position than that in other G_i_-coupled GPCRs, indicating a more vertical orientation in binding. The nearly identical structures with significant differences collectively contribute to a deeper understanding of the structural basis of ET_A_R and ET_B_R activation, their interactions with various G proteins, and the details of the ligand binding interface.

## Materials and methods

### Expression and purification of ET_B_R

We used a previously described human ET_B_R construct with cleavable N- and C-terminal tags. The N-terminus was modified to include the hemagglutinin signal peptide followed by a Flag tag. Rhinovirus 3C protease recognition site (LEVLFQGP) was introduced between G57 and L66. The C-terminus was truncated at S407; three cysteine residues were mutated to alanine (C396A, C400A, and C405A), as described^20^; and fused with an EGFP-HiS9 tag^22^, following rhinovirus 3C protease recognition site. The R124^1.55^Y mutation was introduced to increase thermostability^21^. The resulting construct was introduced into the pFastBac vector. Recombinant baculovirus was prepared using the Bac-to-Bac baculovirus expression system (Invitrogen). *Spodoptera frugiperda* Sf9 insect cells (Invitrogen) were infected with the virus at a cell density of 3.0–4.0 ×10^6^ cells/mL in Sf900 II medium and cultured for 48 h at 27 °C. To purify ET_B_R, harvested cells were lysed with hypotonic lysis buffer (20 mM HEPES [pH 7.5], 0.1 μM ET-1, and protease inhibitors) and centrifuged at 30,000 *×g* for 20 min. The pellet was homogenized with a Dounce homogenizer in a solubilization buffer (1% lauryl maltose neopentyl glycol (LMNG, Anatrace), 0.1% cholesteryl hemisuccinate (CHS, Sigma-Aldrich), 20 mM HEPES [pH 7.5], 200 mM NaCl, 20% glycerol, 0.2 μM ET-1, and protease inhibitors) and solubilized for 1 h at 4 °C. The insoluble cell debris was removed by centrifugation (30,000 *×g*, 20 min), and the supernatant was mixed with TALON cobalt resin (Clontech) for 2 h at 4 °C. The resin was collected in an open glass column, washed with 10 column volumes of wash buffer I (0.01% LMNG, 0.001% CHS, 20 mM HEPES [pH 7.5], 500 mM NaCl, 20% glycerol, and 10 mM imidazole), washed with 5 column volumes of wash buffer II (0.01% LMNG, 0.001% CHS, 20 mM HEPES [pH 7.5], 100 mM NaCl, 10% glycerol, and 10 mM imidazole), and eluted in wash buffer II supplemented with 250 mM imidazole. The eluate was concentrated, mixed with ET-1 to 1 μM, and dialyzed against a buffer containing 0.01% LMNG, 0.001% CHS, 20 mM HEPES (pH 7.5), 100 mM NaCl, 10% glycerol, 0.1 mM TECP, and His-tagged rhinovirus 3 C protease (made in-house) overnight at 4 °C. Following the cleavage of the N-terminus and EGFP–His10 by His-tagged 3 C protease, the sample was mixed with TALON resin for 1 h at 4 °C to remove cleaved EGFP–His10. The ET_B_R-containing flow-through was concentrated and purified by size-exclusion chromatography on a Superdex 200 Increase 10/300 GL gel-filtration column (Cytiva) in a final buffer (100 mM NaCl, 20 mM HEPES [pH 7.5], 5% glycerol, 0.01% LMNG, 0.001% CHS and 0.1 μM ET1). Peak fractions were pooled and concentrated to 4–5 mg/mL.

### Expression and purification of heterotrimeric wild-type G_i1_ and DNG_i1_

Wild-type G_i1_ and DNG_i1_ heterotrimers were expressed in Sf9 or *Trichoplusia ni* Hi5 insect cells (Expression Systems) and purified as described^34^. In brief, insect cells were coinfected with two recombinant viruses: one encoding wild-type human Gα_i1_ or DNGα_i1_ containing four mutations (S47N, G203A, E245A, A326A) and another encoding wild-type human Gβ_1_ and Gγ_2_ subunits with a hexa-histidine tag inserted at the amino terminus of the Gβ_1_ subunit. Cultures were collected 48 h after infection. Cells were lysed in hypotonic buffer, and lipid-modified heterotrimeric G_i1_ or DNG_i1_ was extracted in buffer containing 0.7% sodium cholate, 0.01% LMNG–0.001% CHS, 20 mM HEPES (pH 7.5), 100 mM NaCl, 5 mM MgCl_2_, 1 mM TCEP, 50 μM GDP, and protease inhibitors. The soluble fraction was purified using TALON cobalt resin, and the detergent was exchanged from sodium cholate to 0.01% LMNG–0.001% CHS on a column. After elution was complete, the concentrated protein was dialyzed against a buffer containing 20 mM HEPES (pH 7.5), 100 mM NaCl, 1 mM MgCl_2_, 0.1 mM TCEP, 10 μM GDP, 0.01% LMNG, 0.001% CHS, and His-tagged rhinovirus 3 C protease overnight at 4 °C to cleave the N-terminal His-tag. Then, the sample was mixed with TALON resin for 1 h at 4 °C to remove the cleaved His-tag. The flow-through fraction, containing wild-type G_i1_ or DNG_i1_ heterotrimers, was concentrated and purified by size-exclusion chromatography on a Superdex 200 Increase 10/300 GL gel-filtration column in a final buffer (20 mM HEPES [pH 7.5], 100 mM NaCl, 1 mM MgCl_2_, 0.1 mM TCEP, 10 μM GDP, 0.01% LMNG and 0.001% CHS). Peak fractions were pooled and concentrated to approximately 20 mg/mL.

### Expression and purification of scFv16

Single-chain Fab16 (scFv16) was expressed and purified as described^18,24^. In brief, scFv16 tagged with hexa-histidine at the C-terminus was expressed with a signal peptide in Hi5 insect cells using the Bac-to-Bac baculovirus expression system. The scFv16 secreted into the culture medium was purified by Ni-NTA (Qiagen) chromatography, following addition of Tris (pH 8.0) to the culture supernatant. The Ni-NTA eluent was dialyzed against a buffer consisting of 20 mM HEPES (pH 7.5), 100 mM NaCl, 0.1 mM TCEP, and rhinovirus 3 C protease overnight at 4 °C. The sample was mixed with TALON resin for 1 h at 4 °C to remove the cleaved His-tag. The flow-through fraction containing scFv16 was concentrated and purified by gel-filtration chromatography in a final buffer (100 mM NaCl and 20 mM HEPES [pH 7.5]). Peak fractions were pooled and concentrated to approximately 60 mg/mL.

### Purification of the ET_B_R–G_i1_–scFv16 complex

The ET_B_R–G_i1_–scFv16 complex was prepared as described^18,28^. Purified ET_B_R was mixed with a 1.2 molar excess of wild-type or dominant negative G_i1_ heterotrimer. The coupling reaction proceeded at 20–24 °C for 2 h, followed by incubation for 1 h at 4 °C with apyrase and λ-phosphatase (New England Biolabs) together with 1 mM MnCl_2_ and 5 mM MgCl_2_ for the hydrolysis of unbound GDP and dephosphorylation of proteins, respectively. Furthermore, 1.2 molar excess of scFv16 was added to the mixture and incubated for 2 h at 4 °C. The coupling mixture was incubated with 2A5 anti-ET_B_R immunoaffinity resin overnight at 4 °C^35^. Complex-bound resin was first washed in a buffer containing 0.1% LMNG, 0.01% CHS, and 0.0003% glyco-diosgenin (GDN), then washed in gradually decreasing concentrations of LMNG and increasing concentrations of GDN. The complex was eluted in 20 mM HEPES (pH 7.5), 150 mM NaCl, 0.1 mM TCEP, 2 mM EDTA, 5% glycerol, 0.00375% LMNG, 0.000375% CHS, 0.00125% GDN, 0.1 μM ET-1, and 300 μg/mL 2A5 peptide (VPKGDRTAGSPPRTI) at room temperature. Finally, the ET_B_R–G_i1_–scFv16 complex was purified by size-exclusion chromatography on a Superdex 200 Increase 10/300 GL in 20 mM HEPES (pH 7.5), 100 mM NaCl, 0.1 mM TCEP, 0.1 μM ET-1, 0.00075% LMNG, 0.000075% CHS, and 0.00025% GDN. Peak fractions were concentrated to approximately 30 mg/mL for electron microscopy studies.

### Collection of Cryo-EM Data

Proteins for cryo-EM were prepared to ~6 and 4 mg/mL for ET-1-bound ET_B_R–wild-type G_i1_–scFv16 (ET_B_R–WTG_i_) and ET-1-bound ET_B_R–DNG_i1_–scFv16 (ET_B_R–DNG_i_), respectively. Protein solution (3 μL) was applied to glow-discharged holey carbon grids (200 mesh Quantifoil R2/2 molybdenum and 200 mesh Quantifoil R1.2/1.3 copper for ET_B_R–WTG_i_ and ET_B_R–DNG_i_, respectively), blotted, and plunged into liquid ethane at −182 °C using an EM GP2 plunger (Leica, Microsystems, Vienna, Austria) and Vitrobot Mark IV (Thermo Fisher Scientific) for ET_B_R–WTG_i_ and ET_B_R–DNG_i_, respectively. Data were collected at OIST on a Talos Arctica (Thermo Fisher Scientific, Hillsboro, USA) electron microscope at 200 kV, equipped with a Falcon 3 camera (Thermo Fisher Scientific) and at SPring-8 on a CRYO-ARM300 electron microscope (JEOL) at 300 kV, equipped with a K3 camera (Gatan) (Supplementary Figs. 1, 2). An in-column energy filter with a slit width of 20 eV was inserted to acquire movie frames using CRYO-ARM300. Movies were recorded using EPU software (Thermo Fisher Scientific) on a Talos Arctica at a nominal magnification of 92,000× in counting mode and a pixel size of 1.094 Å at the specimen level, with a dose rate of 0.93 e- per physical pixel per second. Exposure time was 51.3 s, resulting in an accumulated dose of 40 e- per Å^2^. Each movie included 40 fractioned frames. The movies were recorded using SerialEM^36^ and JAFIS Tool version 1 (JEOL) on a CRYO-ARM300 at nominal magnifications of 60,000× and 100,000× in counting mode. The AI detection of each center hole position was performed using yoneoLocr, which prevented any stage alignment failures^37^. The pixel sizes at the specimen level were 0.816 and 0.507 Å for magnifications of 60,000× and 100,000×, with dose rates of 8.3 and 3.4 e- per physical pixel per second, resulting in an accumulated dose of ~76 and ~65 e- per Å^2^ for 6.1 s and 4.9 s exposures, respectively. Each movie included 61 fractioned frames.

### Image processing

All stacked frames were motion corrected with MotionCor2^38^. Defocus was estimated using CTFFIND4^39^. All the particles picked using crYOLO^40^ were analyzed with RELION 3.1^41^ and selected by 2D classification (Table 1, Supplementary Figs. 1, 2). The initial 3D model was generated in RELION, and the particles were divided into four classes by 3D classification, resulting in only one good class. The 3D auto-refinement produced a map, after contrast transfer function refinement, Bayesian polishing, masking, and postprocessing. Particle projections were subjected to subtraction of the detergent micelle density followed by 3D auto-refinement, yielding a final map with resolutions of 4.61, 3.21, and 3.62 Å for ET_B_R–WTG_i_, ET_B_R–DNG_i_, and ET_B_R after focused 3D classification^42^, respectively, according to the gold-standard Fourier shell correlation using a criterion of 0.143 (Supplementary Figs. 3–5 for ET_B_R–WTG_i_, ET_B_R–DNG_i_, and ET_B_R, respectively)^36^. Local resolution maps were calculated using RELION.

### Model building and refinement of the ET_B_R–G_i1_ complex

The atomic models of ET-1 bound ET_B_R (PDB ID: 5GLH) and G_i_–scFv (PDB ID: 6OS9) were fitted to cryo-EM maps of ET_B_R–WTG_i_ and ET_B_R–DNG_i_, respectively, using Chimera^43^. Atomic model building was performed using COOT^44^. The manually modified model was refined in real space on PHENIX^45^, and the COOT/PHENIX refinement was iterated until the refinements converged. Finally, statistics calculated using MolProbity^46^ were checked. Figures were drawn using the Pymol Molecular Graphic System (Schrödinger)^47^, UCSF Chimera^43^, and UCSF ChimeraX^48^.

### MD simulations

The intracellular loop between TM5 and TM6 (residues 302–311) of ET_B_R and α-helical domain of Gα_i_ (residues 56–181, 234–240), which are missing in the cryo-EM structure, were modeled using modeller 9.24^49^. The X-ray crystallographic structures of the D2 dopamine receptor–G_i_ complex (PDB ID: 6VMS) and rhodopsin–G_i_ complex (PDB ID: 6CMO) were used as templates for modeling the intracellular loop of ET_B_R (Supplementary Fig. 12) and the α-helical domain of Gα_i_, respectively. The structure of ET-1-bound ET_B_R–G_i_ was embedded in a solvated 1-palmitoyl-2-oleoyl-*sn*-glycero-3-phosphocholine (POPC) bilayer using the CHARMM-GUI server^50^. The protein structure was protonated using the default settings of the CHARMM-GUI server. The system was composed of 453 POPC molecules, 64,293 water molecules, and 0.15 M K^+^/Cl^−^ ions adjusted to neutralize the net charge of the entire system (Supplementary Table 6). The CHARMM36m force field^51,52^ was used for proteins, ions, and POPC molecules^53^. The TIP3P model^54^ was used for water. Energy minimization and equilibration were performed using the CHARMM-GUI protocol with additional distance restraints between the hydrogen-bond donor and acceptor atoms found in the cryo-EM structure. The parameters for the distance restraints were *r*_0_ = 0 nm, *r*_1_ = 0.3 nm, *r*_2_ = 0.4 nm, and *k* = 4000 kJ mol^−1^ nm^−2^. Then, additional three-step equilibrations were performed with decreasing force constant. Simulations of 50-, 30-, and 20-ns were performed with *k* = 4000, 1000, and 200 kJ mol^−1^ nm^−2^, respectively. After equilibrium simulations, a production run was performed in the constant-*NPT* ensemble for 500 ns. The temperature was maintained at 303.15 K using the Nose–Hoover thermostat^55,56^ with a coupling constant of 1.0 ps. The pressure was maintained at 1.0 bar using a Parrinello–Rahman barostat^57^ with a coupling constant of 5.0 ps. Electrostatic interactions were calculated using the particle mesh Ewald method^58^ with a real space cutoff of 1.2 nm. Van der Waals interactions were calculated with a modified Lennard–Jones potential, where the force was smoothly switched to zero between 1.0 and 1.2 nm. The lengths of the bonds involving hydrogen atoms were constrained using the LINCS algorithm^59,60^ to allow for the use of a time step of 2 fs. The simulations were repeated three times with different initial velocities. All simulations were performed using GROMACS 2022.4^61^.

Probabilities of hydrogen-bond formation in the MD simulations were calculated using the “gmx hbond” tool with default settings. To calculate the density of water molecules, each snapshot of the trajectories was translated and rotated to superpose Cα atoms of ET_B_R on the corresponding atoms of the initial structure. A cubic grid with a spacing of 0.4 Å was then created. Water density (*ρ*_*i*_) at grid point *i* was calculated as follows:

$${\rho }_{i}=\frac{1}{T{V}_{r}}{\sum}_{t=1}^{T}{\sum}_{j=1}^{N}H\left(r-\left|{{{{\bf{x}}}}}_{j,t}-{{{{\bf{c}}}}}_{i}\right|\right),$$

where *T* is the number of snapshots in the trajectories, *N* is the number of water molecules in the system, *V*_*r*_ is the volume of a sphere with radius *r* (*r* = 1 Å), **x**_*j,t*_ represents the coordinates of the oxygen atom of the *j*-th water molecule of the *t*-th snapshot, **c**_*i*_ is the coordinate of the grid point *i*, and *H*(*x*) is the Heaviside step function.

### NanoBiT G-protein dissociation assay

G_i_ activation was measured using a NanoBiT G-protein dissociation assay^7^, in which heterotrimeric G-protein dissociation catalyzed by GPCR was monitored using a NanoBiT system (Promega). A large fragment (LgBiT) of NanoBiT luciferase was inserted into Gα_i1_, and a small fragment (SmBiT) was N-terminally fused to a C68S-mutated Gγ_2_. The amino acid sequences of the NanoBiT G-protein constructs used in this study are identical to those in Inoue et al. ^7^. The genes coding for the NanoBiT G-protein constructs, untagged Gβ_1_ construct, and Flag-tagged ET_B_R were synthesized and cloned into pCAG vectors (provided by Dr. Jun-ichi Miyazaki at Osaka University, Japan) or pcDNA3.1 expression plasmid by GenScript. Mixtures of plasmids prepared for transfection of HEK293A cells (Thermo Fisher Scientific) were prepared as described^7^. HEK293A cells were seeded in a 6-well culture plate at a concentration of 2 ×10^5^ cells/mL (2 mL per well) one day before transfection. Transfection solution was prepared by combining 4 μL (per well hereafter) of polyethylenimine solution (Polysciences; 1 mg/mL) and a plasmid mixture consisting of 100 ng LgBiT-inserted Gα subunit (Gα_i1_), 500 ng Gβ_1_, 500 ng C68S-mutant SmBiT-fused Gγ_2_, and 200 ng wild-type or mutant ET_B_R in 200 μL of Opti-MEM (Thermo Fisher Scientific). After 1-day incubation, transfected cells were collected with 0.5 mM EDTA-containing Dulbecco’s PBS (D-PBS), centrifuged, and suspended in 2 mL of Hank’s Balanced Salt Solution containing 0.01% bovine serum albumin (BSA; fatty-acid-free grade; SERVA) and 5 mM HEPES (pH 7.4) (assay buffer). The cell suspension was dispensed into a white 96-well plate (Greiner Bio-one) at a volume of 80 μL per well and loaded with 20 μL of 50 μM coelenterazine (Carbosynth) diluted in the assay buffer. After 2-h incubation at room temperature in the dark, baseline luminescence was measured (GloMax Navigator, Promega). A range of ET-1 solutions (20 μL of 0–6 × 10^−6^ M) were added and incubated for 3–5 min at room temperature before the second measurement. Luminescence counts were normalized to the initial count, and fold-change signals over vehicle treatment were used to evaluate the G-protein dissociation response. The G-protein activation signals were fitted to a 3-parametric concentration–response curve (GraphPad Prism 9.4), and pEC_50_ values and span values (“Top”–“Bottom”) as *E*_max_ were obtained.

### GloSensor cAMP assay

G_s_ activation was measured by the GloSensor cAMP accumulation assay, in which ET_B_R-induced cAMP accumulation was assayed in cells transiently expressing a biosensor variant, with a cAMP binding domain fused to a luciferase mutant, according to the manufacturer’s instructions (Promega). HEK293A cells were seeded in a 6-well culture plate at a density of 2.5 ×10^5^ cells/mL (2 mL per well) one day before transfection. The cells were transfected with a mixture of pGloSensor cAMP 22 F plasmid (1.5 μg per well) and pCAG expression plasmid encoding ET_B_R or mutant receptors (0.5 μg per well) using 6 μL of FuGENE HD transfection reagent (Promega) in 200 μL of Opti-MEM I reduced serum medium (Thermo Fisher Scientific). After 24 h of incubation, the transfected cells were harvested with 0.5 mM EDTA-containing D-PBS, centrifuged, and suspended in 2 mL of CO_2_-independent medium containing 10% FBS (Invitrogen). The cell suspension was dispensed into a white 96-well plate at a volume of 80 μL per well and loaded with 20 μL of 5 mM D-luciferin in CO_2_-independent medium containing 10% FBS. After 2 h incubation at room temperature in the dark, baseline luminescence was measured (GloMax Navigator, Promega). Varying concentrations of ET-1 solution (20 μL of 0–6 ×10^−6^ M) were added and incubated for 5 min at room temperature before the second measurement. Luminescence counts were normalized to the initial count. To evaluate the G_s_-activated response, fold-change signals over vehicle treatment were represented as percentage of wild-type *E*_max_. The activation signals were fitted to a three-parametric concentration–response curve (GraphPad Prism 9.4), and pEC_50_ and relative *E*_max_ values were obtained. Although a slight decrease was observed in the baseline without ET_B_R expression plasmid (vehicle only) and increasing ET-1 concentration, we did not use phosphodiesterase inhibitors and pertussis toxins, because cAMP signals produced by ET_B_R expression were sufficiently high in HEK293 cells (Supplementary Fig. 10e).

### [^125^I]ET-1 binding assay

In the NanoBiT G-protein dissociation assay and the cAMP accumulation assay, one quarter of the transfected cells were separately frozen in liquid nitrogen and stored at −80 °C. The numbers of wild-type or mutant ET_B_Rs expressed in the transfected cells were monitored by residual [^125^I]ET-1 binding activity, reflecting correctly folded ET_B_Rs. A single-point binding assay using hydroxyapatite resin was performed as described^21^. Briefly, transfected cells were suspended in 50–100 μL of binding buffer containing 50 mM sodium phosphate buffer (pH 7.5), 2 mM MgCl_2_, 0.1% BSA, and 0.1% digitonin. Then, 0.5–2 μL of samples (1.5–6 μg total protein) were incubated with approximately 150 pM [^125^I]ET-1 (PerkinElmer) in 50 μL binding buffer at room temperature for 30 min. Hydroxyapatite resin (30 μL, BioRad) in 15% slurry was added to absorb receptor proteins, and the mixtures were centrifuged at 2000 rpm for 2 min to remove unbound [^125^I]ET-1. Pelleted resin was washed with 0.3 mL of 50 mM sodium phosphate buffer (pH 7.5), 2 mM MgCl_2_, and 0.1% digitonin and measured using a γ-counter. The count of [^125^I]ET-1 bound in the presence of 100 nM ET-1 was subtracted as a background, which was approximately 10% or less of total binding. Each assay was performed in duplicate three times. Relative expression was represented as wild type 100%.

The apparent dissociation constants (*K*_d_) of ET-1 for wild-type and mutant ET_B_ receptors expressed in HEK293A cell membrane were measured using saturation binding assays with [^125^I]ET-1. The cell membranes containing ET_B_ receptors were incubated with eight different concentrations of [^125^I]ET-1 ranging from 2.0 to 200 pM in 50 μl of 50 mM HEPES-NaOH, pH 7.5, 10 mM MgCl_2_ (Mg-HEPES) buffer containing 0.1% BSA at 37 °C for 2 h. Binding reactions were terminated by dilution with cold Mg-HEPES, then were filtered onto glass fiber filters in 96-well plates (multiscreen HTS FB, Merck Millipore) pretreated with 0.1% BSA in Mg-HEPES, to separate the unbound [^125^I]ET-1. After three washes with cold Mg-HEPES, the radioactivity captured by the filters was counted using a γ-counter. The non-specific binding of [^125^I]ET-1 in each reaction was assessed by including 100 nM ET-1 in the same reaction. Assays were performed in duplicate three times and analyzed by fitting to a one-site binding equation (total and nonspecific) using GraphPad Prism 9.4.

### Statistics and reproducibility

NanoBiT G-protein dissociation assay and GloSensor cAMP assay were analyzed using GraphPad Prism 9.4 (GraphPad) and are presented as mean ± standard error of the mean (SEM) from three to five independent experiments conducted in duplicate or triplicate. Statistical analyses were performed using Prism 9.4 (GraphPad) with one-way analysis of variance followed by Dunnett’s multiple comparison of means test or Student’s *t* test. Significance levels in statistical differences are indicated as (****p < 0.0001, ***p < 0.001, **p < 0.01, *p < 0.05 vs. WT).

### Reporting summary

Further information on research design is available in the Nature Portfolio Reporting Summary linked to this article.

## Supplementary information

Peer review file

Supplementary Information

Description of additional supplementary files

Supplementary Data

Reporting Summary

## Data Availability

The map and model generated in this study have been deposited in the EMDB and PDB with accession codes: EMD-38741 and PDB-8XWQ for the ET-1-bound ET_B_R-wild-type G_i1_-scFv16 complex, EMD-38740 and PDB-8XWP for the ET-1-bound ET_B_R–DNG_i1_-scFv16 complex and EMD-60404 and PDB-8ZRT for the focused 3D refinement of ET_B_R in the ET-1-bound ET_B_R–DNG_i1_-scFv16 complex. All other study data including uncropped gel images are included in the article and/or Supplementary Data.
