# Supplementary material for: Structure of endothelin ETB receptor–Gi complex in a conformation stabilized by unique NPxxL motif
Source: Commun Biol. 2024 Oct 16;7:1303. doi: 10.1038/s42003-024-06905-z (PMC11484851; doi:10.1038/s42003-024-06905-z)
Supplement: Supplementary file 2 — Supplementary Information [file 42003_2024_6905_MOESM2_ESM.pdf]

SUPPLEMENTARY INFORMATION

Structure of endothelin ET_B receptor-Gi complex in a conformation stabilized by the unique NPxxL motif

Kazutoshi Tani, Saori Maki-Yonekura, Ryo Kanno, Tatsuki Negami, Tasuku Hamaguchi, Malgorzata Hall, Akira Mizoguchi, Bruno M. Humbel, Tohru Terada, Koji Yonekura, Tomoko Doi

Corresponding authors: K. Tani and T. Doi

E-mail: ktani@ccs.tsukuba.ac.jp; doi.tomoko.8n@kyoto-u.jp

This file contains:

(1) Supplementary Tables 1 to 6

(2) Supplementary Figures 1 to 13

(3) Supplementary References

(4) Supplementary Figure 14 (The uncropped gel images)

Supplementary Table 1: ET-1-mediated activation of WT and mutant ET_B receptors in the G_i dissociation assay. Data were collected through the G_i dissociation assay in parallel. pEC_{50} and E_{max} estimates represent average value and standard error of the mean (SEM), respectively, from three to five independent experiments performed in duplicate or triplicate. “N.D.” denotes cases where no activity was detected. [a), b), and c)]; Statistical differences from wild type (100%), wild type (50%), and wild type (35%) were analyzed using ANOVA with Dunnet’s multiple comparison of means test, respectively. Significance levels are indicated as (****$p < 0.0001$, ***$p < 0.001$, **$p < 0.01$, *$p < 0.05$ vs. WT).

(a) Related to Fig. 2f.

**ET_BR constructs**	**pEC_{50}**	**E_{max}**	**E_{max}, %WT**	**n**	**relative ET_BR expression**	**n**
WT (100%)	9.66 ± 0.06	0.30 ± 0.010	100 ± 3.1	4	100	9
D198A (3.49)	9.86 ± 0.04	0.30 ± 0.007[b)]	99.7 ± 2.6	3	53.1 ± 4.8	6
R199A (3.50)	N.D.	N.D.	N.D.	3	83.2 ± 6.0	7
Y293F (5.58)	9.39 ± 0.18	0.05 ± 0.005****[a)]	5.5 ± 1.7	3	75.6 ± 9.3	5
N382A (7.49)	9.53 ± 0.23	0.04 ± 0.005****[b)]	2.5 ± 1.8	3	52.9 ± 1.7	5

(b) Related to Fig. 2g.

**ET_BR constructs**	**pEC_{50}**	**E_{max}**	**E_{max}, %WT**	**n**	**relative ET_BR expression**	**n**
WT (100%)	9.13 ± 0.05	0.26 ± 0.007	100.0 ± 3.0	4	100	3
L386Y (7.53)	9.70 ± 0.16	0.04 ± 0.004****[c)]	15.4 ± 1.5	4	39.8 ± 4.8	3
L386A (7.53)	9.89 ± 0.17	0.03 ± 0.003****[c)]	11.5 ± 1.2	4	34.3 ± 6.0	3
L386N (7.53)	N.D.	N.D.	N.D.	3	29.0 ± 2.3	3
L386V (7.53)	9.15 ± 0.11	0.11 ± 0.006****[b)]	33.5 ± 2.5	3	57.5 ± 4.4	3
L386I (7.53)	8.84 ± 0.07	0.15 ± 0.005****[a)]	49.6 ± 2.1	4	99.1 ± 4.9	3

(c) Related to Fig. 5a.

ET_BR constructs	pEC_{50}	E_{max}	E_{max}, %WT	n	relative ET_BR expression	n
WT (100%)	9.66 ± 0.06	0.30 ± 0.010	100 ± 3.1	4	100	9
WT (50%)	9.67 ± 0.08	0.24 ± 0.011	76.5 ± 4.0	3	66.0 ± 29.2	3
WT (35%)	9.70 ± 0.07	0.20 ± 0.008	62.9 ± 2.9	3	38.2 ± 12.4	3
N134A (ICL1)	9.60 ± 0.06	0.18 ± 0.005*[c)]	54.6 ± 2.0	3	35.5 ± 1.5	4
S390A (8.47)	9.43 ± 0.08	0.11 ± 0.005****[c)]	28.8 ± 1.8	3	41.5 ± 3.8	4

(d) Related to Fig. 5b.

ET_BR constructs	pEC_{50}	E_{max}	E_{max}, %WT	n	relative ET_BR expression	n
WT (100%)	9.66 ± 0.05	0.29 ± 0.008	100 ± 3.2	5	100	11
M296A (5.61)	9.05 ± 0.08	0.13 ± 0.005****[a)]	36.2 ± 2.0	3	91.5 ± 1.9	3
M300A (5.65)	9.18 ± 0.11	0.15 ± 0.009****[a)]	46.9 ± 3.3	3	109.7 ± 14.0	3
V325A (6.37)	9.39 ± 0.08	0.20 ± 0.009****[a)]	63.5 ± 3.3	3	89.5 ± 11.9	3

(e) Related to Fig. 5c.

ET_BR constructs	pEC_{50}	E_{max}	E_{max}, %WT	n	relative ET_BR expression	n
WT (100%)	9.66 ± 0.05	0.29 ± 0.008	100 ± 3.2	5	100	11
H314A (6.26)	9.59 ± 0.06	0.27 ± 0.009	90.2 ± 3.5	3	62.7 ± 6.4	3
R318A (6.30)	9.31 ± 0.06	0.26 ± 0.009	88.2 ± 3.3	3	102.4 ± 16.2	3
T324A (6.36)	9.53 ± 0.06	0.23 ± 0.008[b)]	77.3 ± 3.0	3	71.4 ± 2.2	3
V389A (7.56)	9.60 ± 0.06	0.28 ± 0.009	96.3 ± 3.6	3	69.6 ± 5.5	3
K391A (8.48)	9.59 ± 0.05	0.27 ± 0.008	91.2 ± 2.9	3	61.9 ± 10.6	3

(f) Related to Fig. 5d.

$G\alpha_i$ constructs	pEC_{50}	E_{max}	E_{max}, %WT	n
WT	9.32 ± 0.05	0.30 ± 0.008	100 ± 3.0	3
F354A (H5.26)	9.25 ± 0.04	0.26 ± 0.006**	87.8 ± 2.2	3
L353A (H5.25)	9.48 ± 0.15	0.05 ± 0.004****	7.8 ± 1.6	3
G352A (H5.24)	9.02 ± 0.05	0.20 ± 0.005****	61.8 ± 1.9	3
C351A (H5.23)	9.20 ± 0.04	0.26 ± 0.005***	87.0 ± 1.9	3
D350A (H5.22)	9.40 ± 0.04	0.28 ± 0.005	92.2 ± 2.1	3
N347A (H5.19)	9.43 ± 0.04	0.29 ± 0.007	96.5 ± 2.5	3
K345A (H5.17)	9.06 ± 0.06	0.16 ± 0.005****	48.9 ± 1.9	3
D341A (H5.13)	9.51 ± 0.04	0.28 ± 0.006	92.1 ± 2.4	3

Supplementary Table 2: ET-1-induced activation of WT and mutant ET_B receptors in the G_s-mediated cAMP accumulation assay. Data were collected through the cAMP accumulation assay in parallel. pEC_{50} and E_{max} estimates represent average value and standard error of the mean (SEM), respectively, from three to five independent experiments performed in duplicate. "N.D." is used to denote cases where no activity was detected. [a), b), and c)]; Statistical differences from wild type (75%), wild type (50%), and wild type (25%) were analyzed using ANOVA with Dunnett's multiple comparison of means test, respectively. [d)]; Statistical difference from wild type (100%) was determined using Student's *t*-test. Significance levels are indicated as (****p < 0.0001, ***p < 0.001, **p < 0.01, *p < 0.05 vs WT).

(a) Related to Supplementary Fig. 10a

ET_BR constructs	pEC_{50}	E_{max}, %WT	n	relative ET_BR expression	n
WT	8.52 ± 0.03	99.9 ± 1.4	5	100	3
N134A (ICL1)	8.79 ± 0.25	20.3 ± 2.2****[a)]	3	80.2 ± 10.4	3
D198A (3.49)	8.95 ± 0.16	48.2 ± 3.5****[a)]	3	73.1 ± 3.4	3
R199A (3.50)	N.D.	N.D.	3	87.5 ± 11.7	3
Y293F (5.58)	N.D.	N.D.	3	90.4 ± 4.4	3
N382A (7.49)	N.D.	N.D.	3	63.9 ± 8.3	3
S390A (8.47)	8.89 ± 0.86	2.9 ± 1.1****[b)]	3	48.0 ± 4.8	3

(b) Related to Supplementary Fig. 10b

ET_BR constructs	pEC_{50}	E_{max}, %WT	n	relative ET_BR expression	n
WT	8.39 ± 0.05	99.7 ± 2.1	3	100	3
L386Y (7.53)	8.52 ± 0.29	4.9 ± 0.6****[c)]	3	37.4 ± 2.0	3
L386A (7.53)	8.23 ± 0.31	2.9 ± 0.4****[c)]	3	33.3 ± 5.2	3
L386N (7.53)	N.D.	N.D.	3	36.1 ± 3.3	3
L386I (7.53)	8.60 ± 0.13	25.8 ± 1.3****[a)]	3	78.0 ± 9.5	3
L386V (7.53)	8.40 ± 0.13	26.3 ± 1.4****[b)]	3	58.6 ± 1.6	3

(c) Related to Supplementary Fig. 10c

ET_BR constructs	pEC_{50}	E_{max}, %WT	n	relative ET_BR expression	n
WT	8.52 ± 0.03	99.9 ± 1.4	5	100	3
M296A (5.61)	N.D.	N.D.	3	93.6 ± 2.3	3
M300A (5.65)	N.D.	N.D.	3	74.7 ± 9.3	3
H314A (6.26)	8.69 ± 0.11	85.9 ± 4.1[b)]	3	49.9 ± 5.3	3
R318A (6.30)	8.31 ± 0.15	17.2 ± 1.0****[a)]	3	79.0 ± 5.2	3
T324A (6.36)	8.54 ± 0.08	39.4 ± 1.2****[d)]	3	91.8 ± 8.6	3
V325A (6.37)	8.74 ± 0.20	8.3 ± 0.7****[b)]	3	50.3 ± 8.5	3
V389A (7.56)	8.80 ± 0.07	33.2 ± 1.1****[b)]	3	63.6 ± 8.8	3
K391A (8.48)	8.59 ± 0.07	69.3 ± 1.9**[a)]	3	67.8 ± 7.5	3

(d) Related to Supplementary Fig. 10d

WT ET_BR	pEC_{50}	E_{max}, %WT	n
100%	8.21 ± 0.09	97.2 ± 3.7	3
75%	8.36 ± 0.08	79.5 ± 2.7	3
50%	8.54 ± 0.11	49.2 ± 2.2	3
25%	8.77 ± 0.16	19.8 ± 1.4	3
0%	N.D.	N.D.	2

(e) Related to Supplementary Fig. 10e

ET_BR constructs	pEC_{50}	E_{max}	n
WT	8.46 ± 0.05	10.15 ± 0.17	3
vehicle	N.D.	N.D.	2

Supplementary Table 3: List of contacts between ET_BR and $G\alpha_i$.

$G\alpha_i$ residue	ET_BR residue	Type of interaction
$L194^{S3.1}$	$I209^{ICL2}$	Nonbonded contact
$T340^{H5.12}$	$W206^{ICL2}$	Nonbonded contact
$D341^{H5.13}$	$H314^{6.26}$	Nonbonded contact
	$R318^{6.30}$	Hydrogen bond
$I343^{H5.15}$	$I209^{ICL2}$	Nonbonded contact
$I344^{H5.16}$	$W206^{ICL2}$	Nonbonded contact
	$V203^{3.54}$	Nonbonded contact
$N346^{H5.18}$	$K210^{ICL2}$	Nonbonded contact
$N347^{H5.19}$	$A202^{3.53}$	Nonbonded contact
	$R208^{ICL2}$	Hydrogen bond
$L348^{H5.20}$	$V203^{3.54}$	Nonbonded contact
	$M300^{5.65}$	Nonbonded contact
$D350^{H5.22}$	$N134^{ICL1}$	Hydrogen bond with peptide backbone
	$K210^{ICL2}$	Hydrogen bond
$C351^{H5.23}$	$P136^{2.39}$	Nonbonded contact
	$R199^{3.50}$	Nonbonded contact
$G352^{H5.24}$	$L386^{7.53}$	Nonbonded contact
	$S390^{8.47}$	Hydrogen bond with peptide backbone
$L353^{H5.25}$	$R199^{3.50}$	Nonbonded contact
	$M296^{5.61}$	Nonbonded contact
	$V321^{6.33}$	Nonbonded contact
	$V325^{6.37}$	Nonbonded contact
	$V389^{7.56}$	Nonbonded contact
$F354^{H5.26}$	$Q317^{6.29}$	Nonbonded contact
	$V321^{6.33}$	Nonbonded contact
	$V389^{7.56}$	Hydrogen bond with peptide backbone
	$S390^{8.47}$	Hydrogen bond with peptide backbone

Supplementary Table 4: Saturation binding using [^{125}I]ET-1 and HEK293A cell membranes expressing each mutant protein. The K_d values are presented as mean ± SEM (pM), representing data from three independent experiments conducted in duplicates.

ET_BR construct	K_d (pM)
WT	16.8 ± 1.7
N134A^{ICL1}	14.0 ± 2.4
D198A$^{3.49}$	14.9 ± 1.0
R199A$^{3.50}$	15.5 ± 0.5
Y293F$^{5.58}$	15.2 ± 0.3
M296A$^{5.61}$	19.1 ± 1.9
M300A$^{5.65}$	17.3 ± 4.0
T324M$^{6.36}$	17.7 ± 3.4
V325A$^{6.37}$	17.3 ± 2.2
N382A$^{7.49}$	15.1 ± 1.1
S390A$^{8.47}$	16.3 ± 1.4

Supplementary Table 5: Frequency of hydrogen bond occurrence in MD simulations.

	run1	run2	run3	all
$D341^{H5.13}$-$R318^{6.30}$	0.969	0.341	0.960	0.757
$N347^{H5.19}$-$R208^{ICL2}$	0.200	0.010	0.005	0.071
$D350^{H5.22}$-$N134^{ICL1}$	0.654	0.419	0.138	0.404
$F354^{H5.26}$-$S390^{8.47}$	0.686	0.987	0.412	0.695
$D147^{2.50}$-$N382^{7.49}$	0.995	0.951	0.078	0.675
$D147^{2.50}$-$S379^{7.46}$	0.999	0.999	0.984	0.994
$R199^{3.50}$-$Y293^{5.58}$	0.631	0.449	0.050	0.377

Supplementary Table 6: System setup of the MD simulations

System	ET-1-bound ET_BR–G_i,<br>453 POPC,<br>64,293 TIP3P water,<br>0.15 M KCl
Total number of atoms	270,932
Simulation box	130 Å × 130 Å × 171 Å
Number of simulations	3

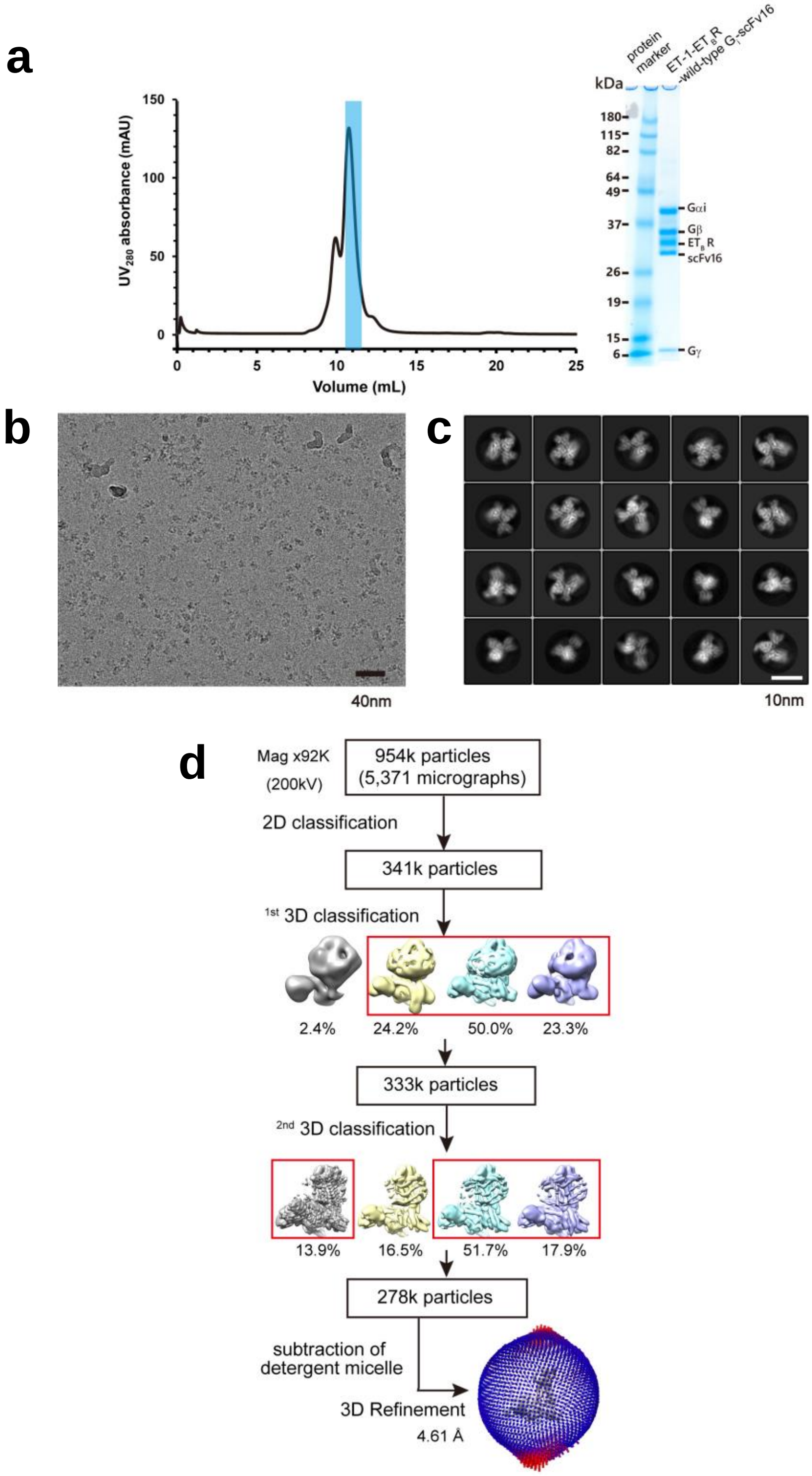

Supplementary Fig. 1: Structure determination of ET-1–ET$_B$R–wild-type G$_i$–scFv16 by cryo-EM. (a) (left) Representative elution profiles of the ET-1–ET$_B$R–wild-type G$_i$–scFv16 complex on a Superdex 200 Increase 10/300 GL gel-filtration column. The used fraction of column volume is indicated by a cyan box. (right) SDS-PAGE of the purified ET-1–ET$_B$R–wild type G$_i$–scFv16 complex. **(b)** A representative micrograph of the ET-1–ET$_B$R–wild type G$_i$–scFv16 complex. **(c)** Representative 2D class averages from micrographs. **(d)** Image processing flow of 3D classification and reconstruction. Angular distribution of reconstructed particles used in the final refinement.

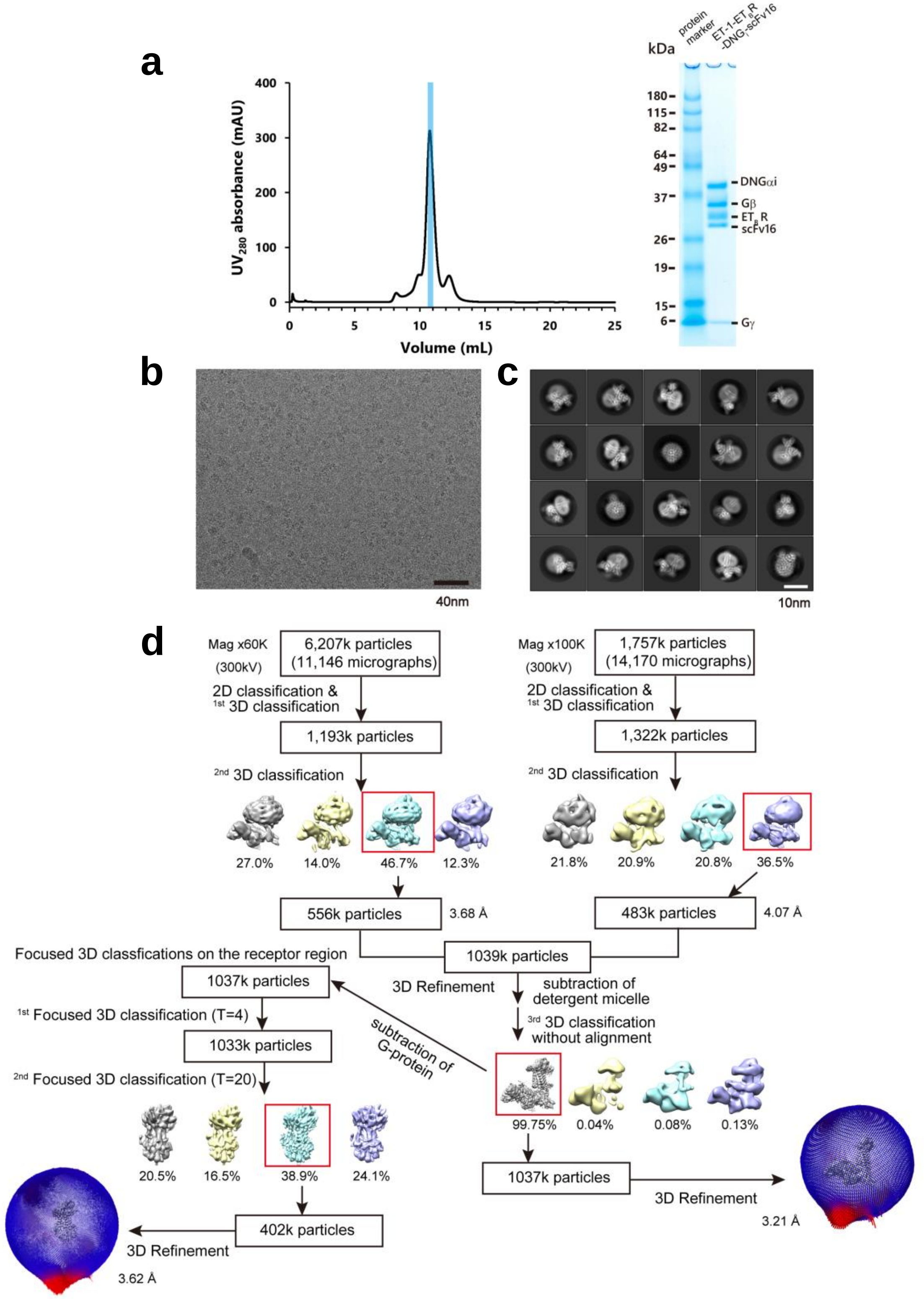

Supplementary Fig. 2: Structure determination of ET-1–ET$_B$R–DNG$_i$–scFv16 by cryo-EM. **(a)** (left) Representative elution profiles of the ET-1–ET$_B$R–DNG$_i$–scFv16 complex on a Superdex 200 Increase 10/300 GL gel-filtration column. The used fraction of column volume is indicated by a cyan box. (middle) SDS-PAGE of the purified ET-1–ET$_B$R–DNG$_i$–scFv16 complex. (right) The uncropped gel image. **(b)** A representative micrograph of the ET-1–ET$_B$R–DNG$_i$–scFv16 complex. **(c)** Representative 2D class averages from micrographs. **(d)** Image processing flow of 3D classification and reconstruction. Angular distribution of reconstructed particles used in the final refinement.

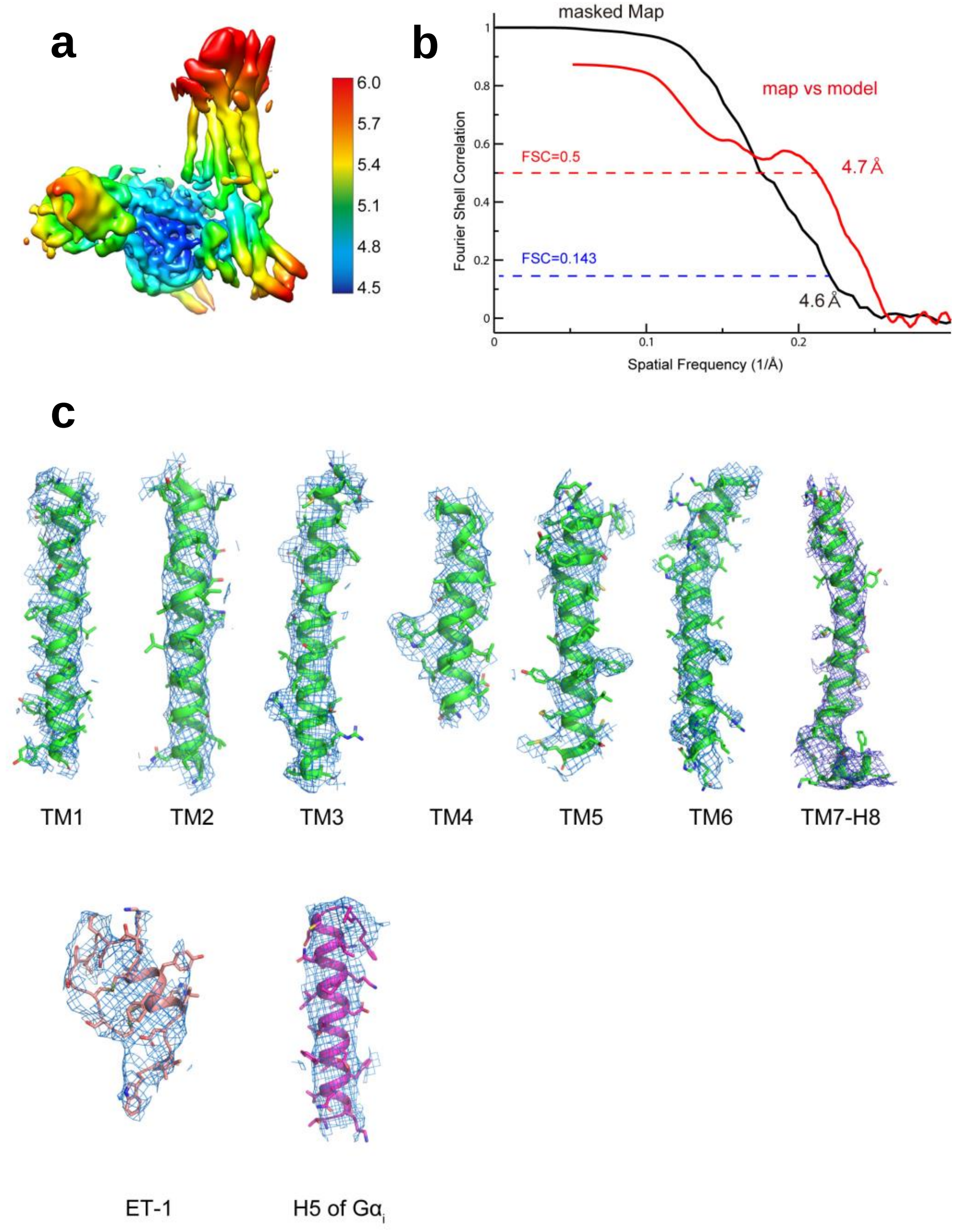

Supplementary Fig. 3: Cryo-EM densities and structural models in the ET-1–ET$_B$R–wild-type G$_i$–scFv16 complex. **(a)** The overall structure of the ET-1 bound ET$_B$R–wild-type G$_i$ complex is represented in rainbow colors according to local resolution (shown in the color bar on the right). **(b)** Fourier shell correlation (FSC) plots of the cryo-EM map (masked: black) and the FSC plot of the model versus the final map (red) are superimposed. Global resolution defined at FSC = 0.143 is 4.61. **(c)** Selected regions of the cryo-EM density superimposed on selected regions of the refined model. The density maps are shown at a contour level of 4.5 σ. The color codes are the same as those in Fig. 1.

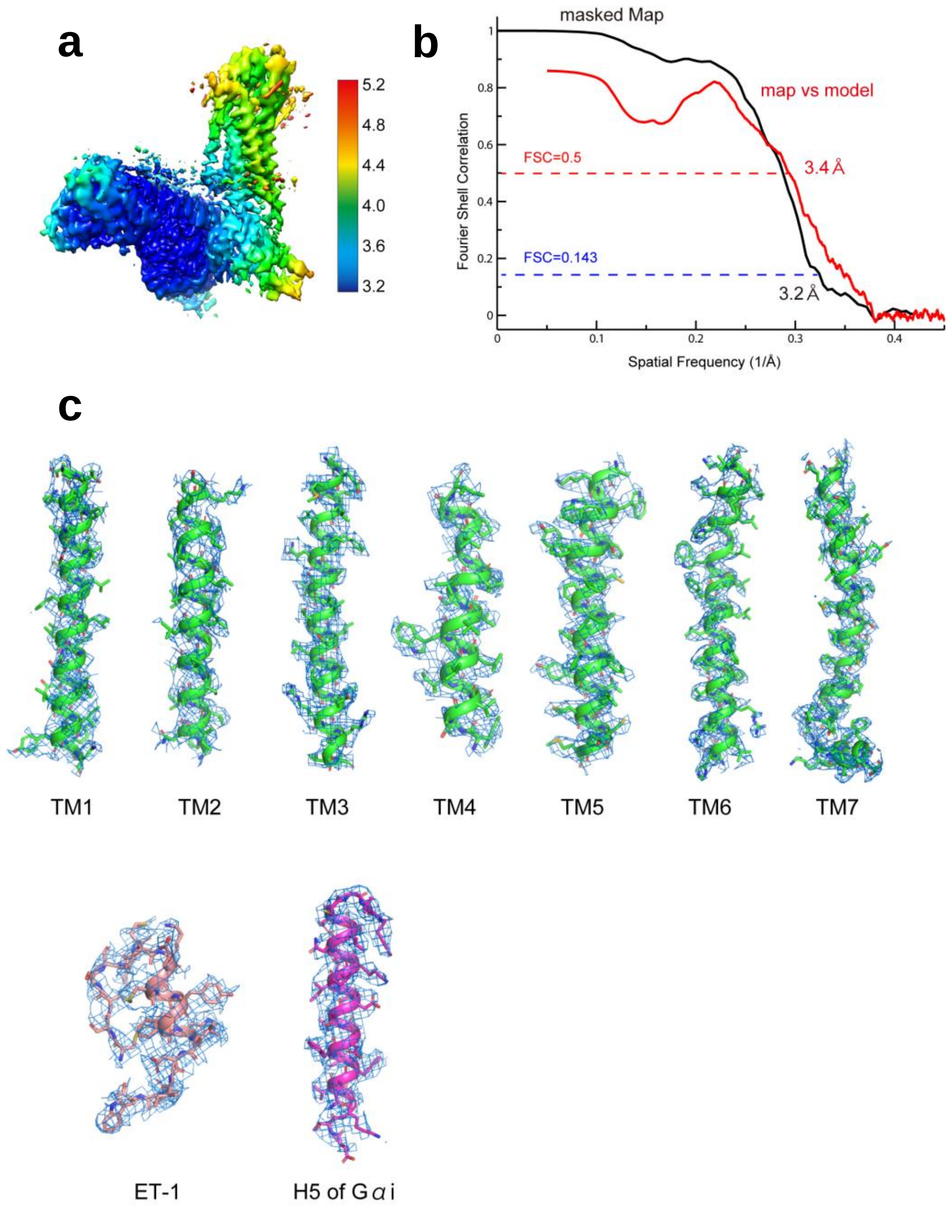

Supplementary Fig. 4: Cryo-EM densities and structural models in the ET-1–ET_BR–DNG_i–scFv16 complex. (a) Overall structure of the ET-1 bound ET_BR–DNG_i complex is represented in rainbow colors according to local resolution (shown in the color bar on the right). **(b)** Fourier shell correlation (FSC) plots of the cryo-EM map (masked: black) and the FSC plot of the model versus the final map (red) are superimposed. Global resolution at FSC = 0.143 is 3.21. **(c)** Selected regions of the cryo-EM density superimposed on selected regions of the refined model. The density maps are shown at a contour level of 4.0 σ. The color codes are the same as those in Fig. 1.

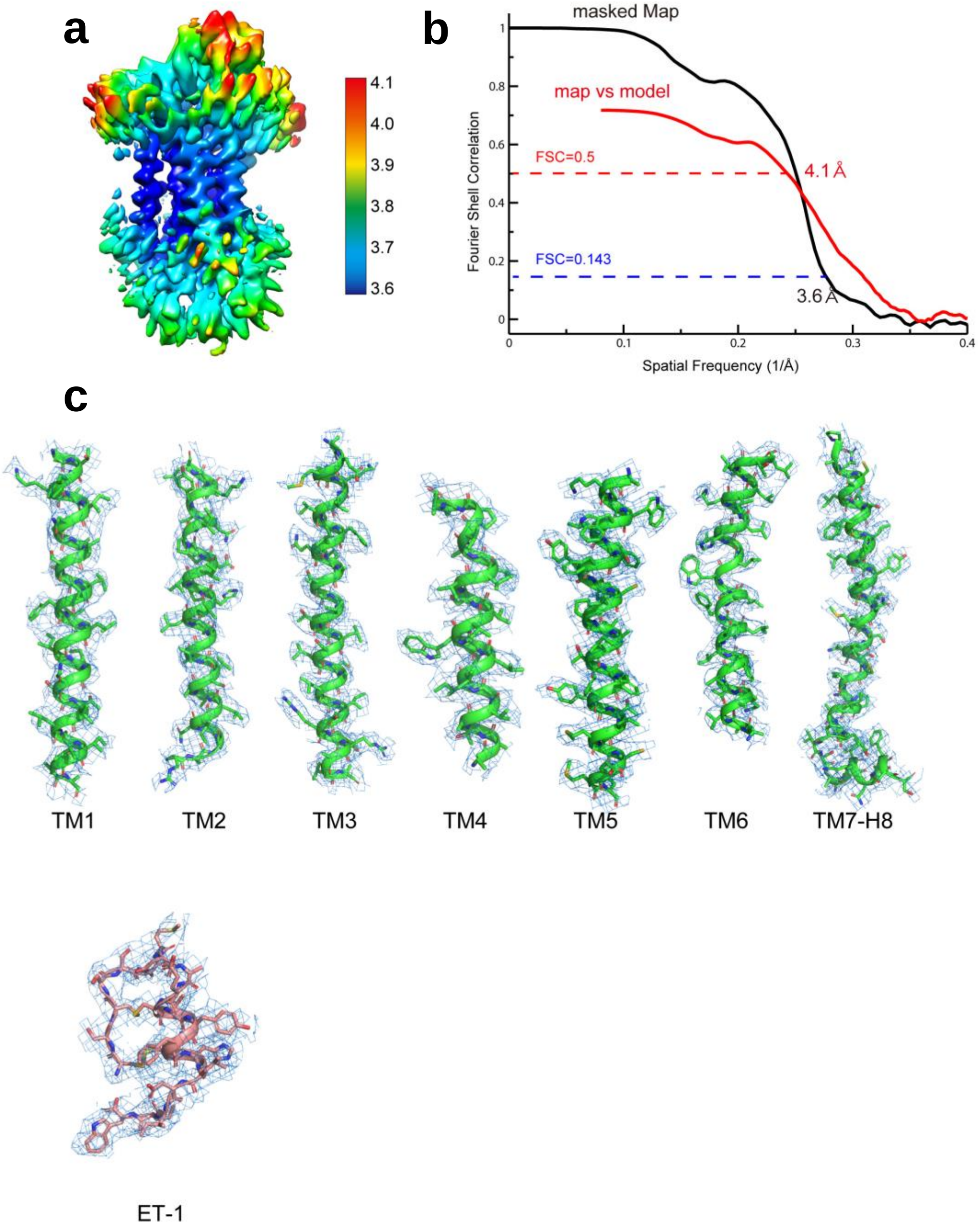

Supplementary Fig. 5: Cryo-EM densities and structural models after the 3D focused refinement on the receptor in ET-1–ET$_B$R complex. (a) Overall structure of the ET-1 bound ET$_B$R complex is represented in rainbow colors according to local resolution (shown in the color bar on the right). **(b)** Fourier shell correlation (FSC) plots of the cryo-EM map (masked: black) and the FSC plot of the model versus the final map (red) are superimposed. Global resolution at FSC = 0.143 is 3.62. **(c)** Selected regions of the cryo-EM density superimposed on selected regions of the refined model. The density maps are shown at a contour level of 4.0 σ. The color codes are the same as those in Fig. 1.

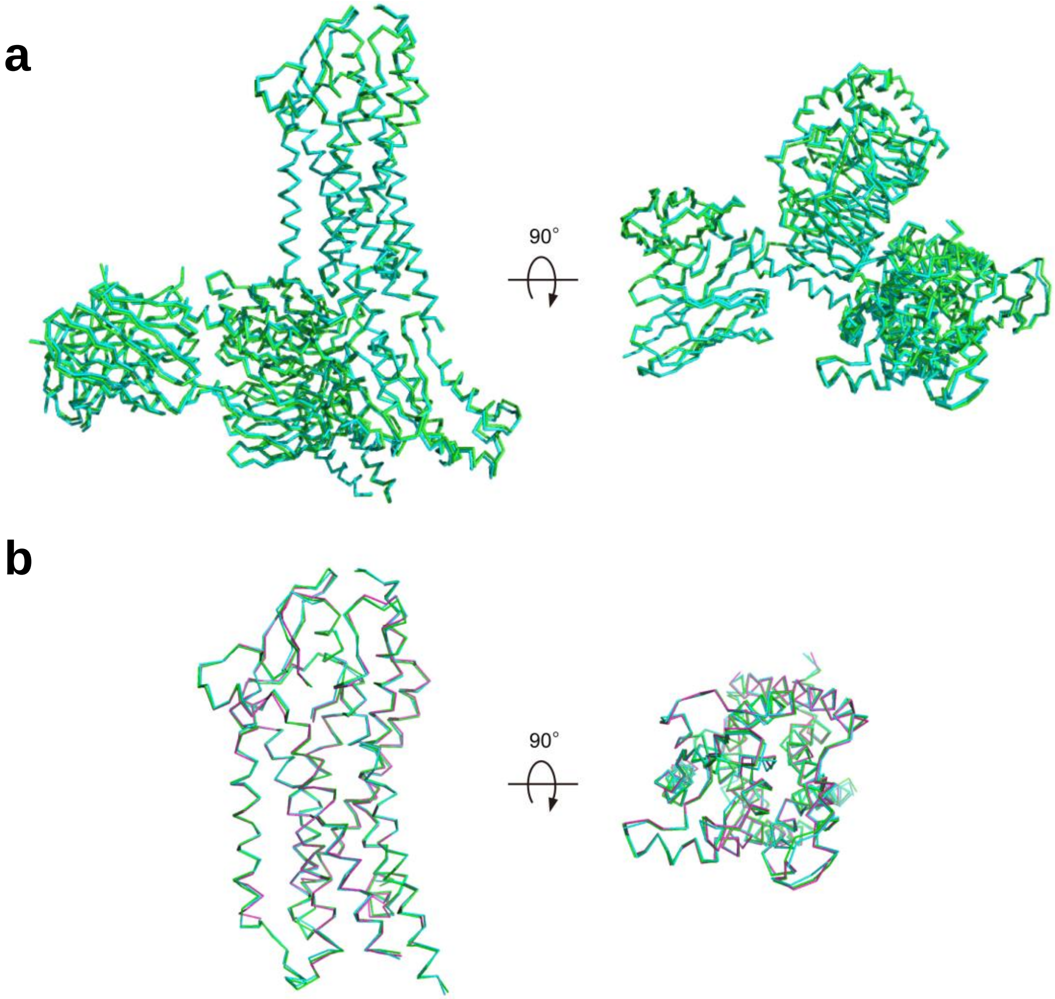

Supplementary Fig. 6: Structural comparison of the ET-1–ET$_B$R–DNG$_i$–scFv16 complex and the ET-1–ET$_B$R–wild-type G$_i$–scFv16 complex. (a) Side and top view displaying the superposition of the ET-1 bound ET$_B$R–DNG$_i$ complex (in green) with the ET-1 bound ET$_B$R–wild-type G$_i$ complex (in cyan). The overall RMSD of the Cα atoms between them is 0.662. (b) Side and top view displaying the superposition of the ET-1 bound ET$_B$R complexes (green for the ET$_B$R–DNG$_i$, cyan for the ET$_B$R–wild-type G$_i$, and magenta for the focused 3D refinement on the receptor in the ET$_B$R–DNG$_i$). The overall RMSD of the Cα atoms between ET-1 bound receptors compared to the reference receptor model in the ET$_B$R–DNG$_i$ are measured at 0.391 for the ET$_B$R–wild-type G$_i$ and 0.364 for the focused 3D refinement of ET$_B$R in the ET$_B$R–DNG$_i$.

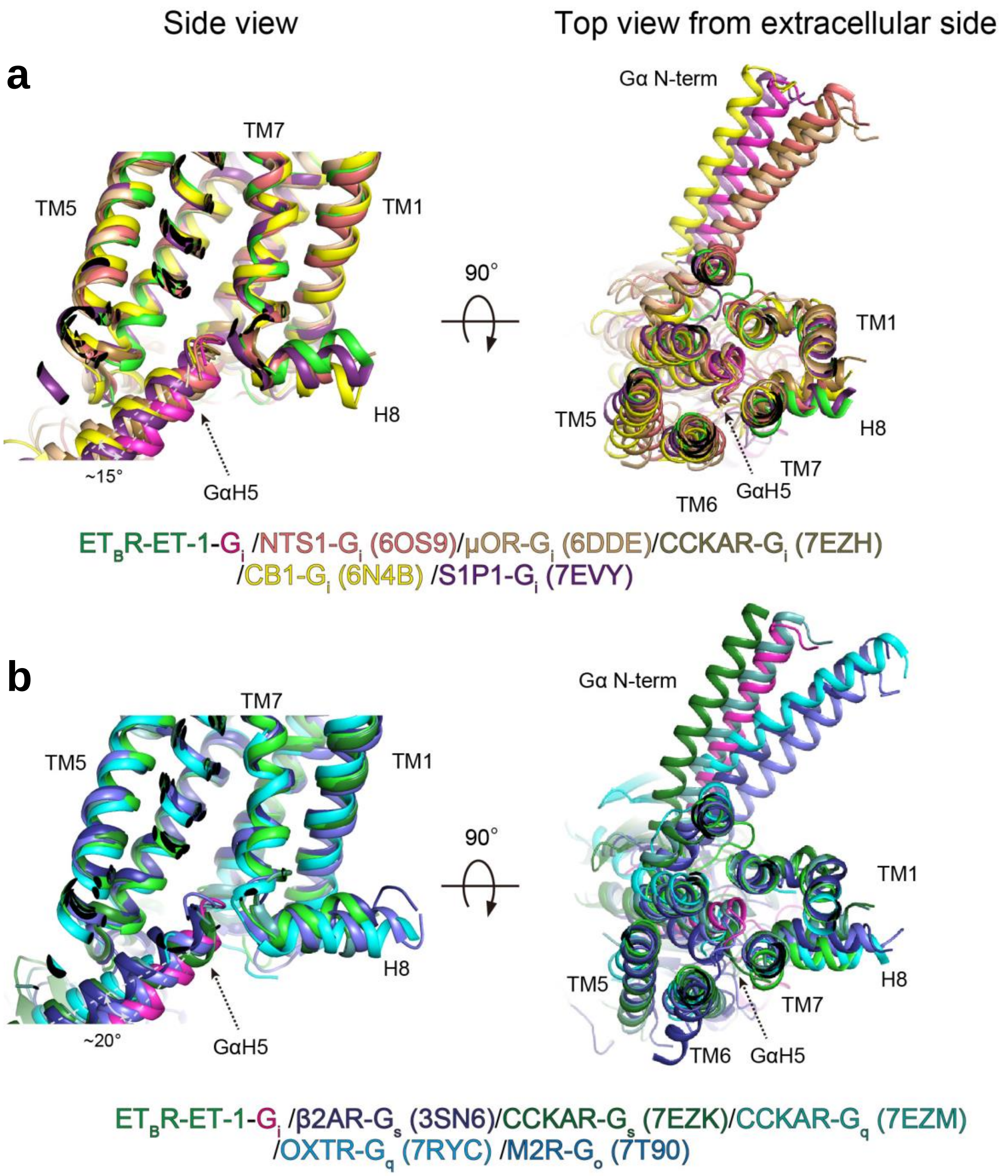

Supplementary Fig. 7: Structural comparison of the ET-1–ET$_B$R–DNG$_i$–scFv16 complex with other GPCR–G complexes. (a) Side and extracellular top views showing the superposition of the ET-1 bound ET$_B$R–DNG$_i$ complex (green and magenta) with other GPCR–G$_i$ complexes. The contact surface area between the receptor and G$_i$ subunit is as follows: 1132 Å^2 for ET$_B$R–DNG$_i$, 1095 Å^2 for NTS1–G$_i$, 1208 Å^2 for μOR–G$_i$, 1146 Å^2 for CCKAR–G$_i$, 910 Å^2 for CB1–G$_i$, and 1426 Å^2 for S1P1–G$_i$. **(b)** Side and extracellular top views showing the superposition of the ET-1 bound ET$_B$R–DNG$_i$ complex (green and magenta) with the GPCR–G$_s$, –G$_q$, and –G$_o$ complexes. The contact surface area between the receptor and G subunit is as follows: 1327 Å^2 for βAR–G$_s$, 1365 Å^2 for CCKAR–G$_s$, 1508 Å^2 for CCKAR–G$_q$, 724 Å^2 for OXTR–G$_q$, and 1038 Å^2 for M2R–G$_o$. Contact surfaces were calculated using AREAIMOL in the CCP4 software suite[S1].

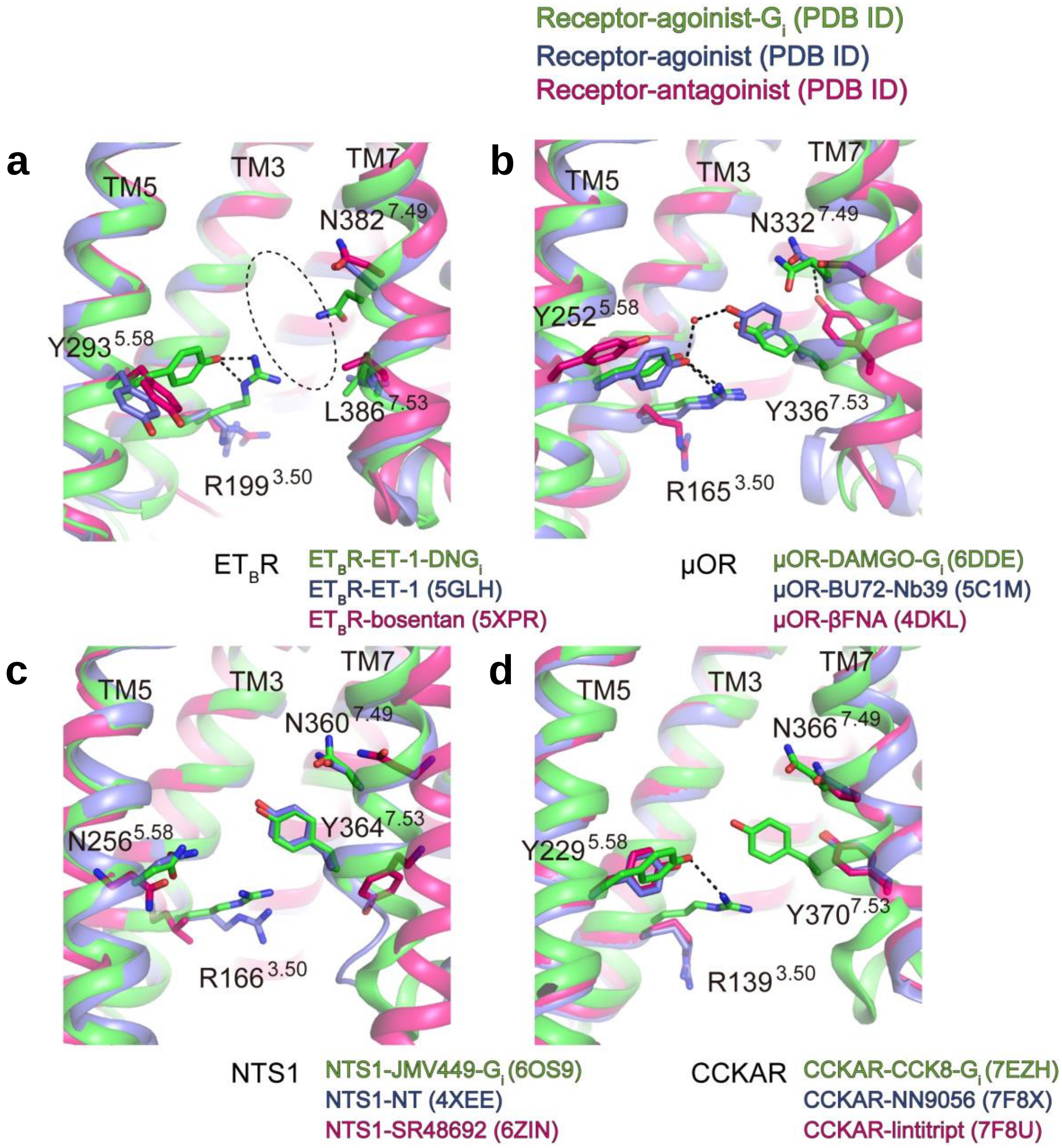

Supplementary Fig. 8: Structural comparison of ET$_B$R with other GPCRs. A comparison of the activation-dependent inward movement of TM7 in ET$_B$R and three GPCRs: μOR–G$_i$, NTS1–G$_i$, and CCKAR–G$_i$. The active state of the N$^{7.49}$P$^{7.50}$xxY$^{7.53}$ motif exhibits a similar conformation but differs from that of the N$^{7.49}$P$^{7.50}$xxL$^{7.53}$ motif, resulting in the creation of a cavity (indicated by a dashed oval in (a)).

a

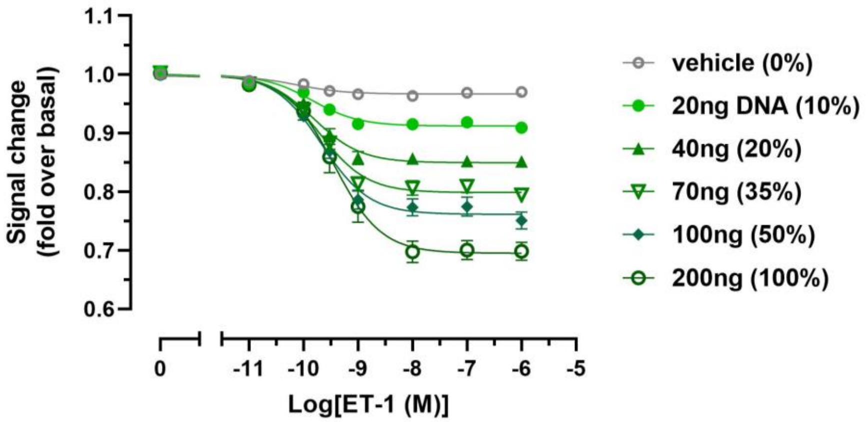

b

WT ET_BR	pEC_{50}	E_{max}	E_{max}, %WT	n	relative ET_BR expression	n
100%	9.45 ± 0.09	0.30 ± 0.015	100 ± 5.5	3	100	3
50%	9.67 ± 0.08	0.24 ± 0.011	76.5 ± 4.0	3	66.0 ± 29.2	3
35%	9.70 ± 0.07	0.20 ± 0.008	62.9 ± 2.9	3	38.2 ± 12.4	3
25%	9.71 ± 0.07	0.17 ± 0.007	52.4 ± 2.5	3	32.1 ± 8.1	3
20%	9.82 ± 0.09	0.15 ± 0.007	44.0 ± 2.6	3	22.4 ± 5.6	3
10%	9.84 ± 0.09	0.09 ± 0.004	20.8 ± 1.6	3	11.8 ± 2.8	3
0%	N.D.	N.D.	N.D.	3	N.D.	4

Supplementary Fig. 9: G_i dissociation assay of ET_BR. (a) Presentation of G_i dissociation assay results for WT ET_BR at reduced levels of expression (%DNA transfected). WT (100%) corresponds to the transfection of 200 ng of wild-type ET_BR expression plasmid for one well of a 6-well plate in G_i dissociation assays, as described in the Methods section. **(b)** pEC_{50} and E_{max} estimates represent the average value and standard error of the mean (SEM), respectively, derived from three independent experiments performed in duplicate. N.D. indicates no detected activity.

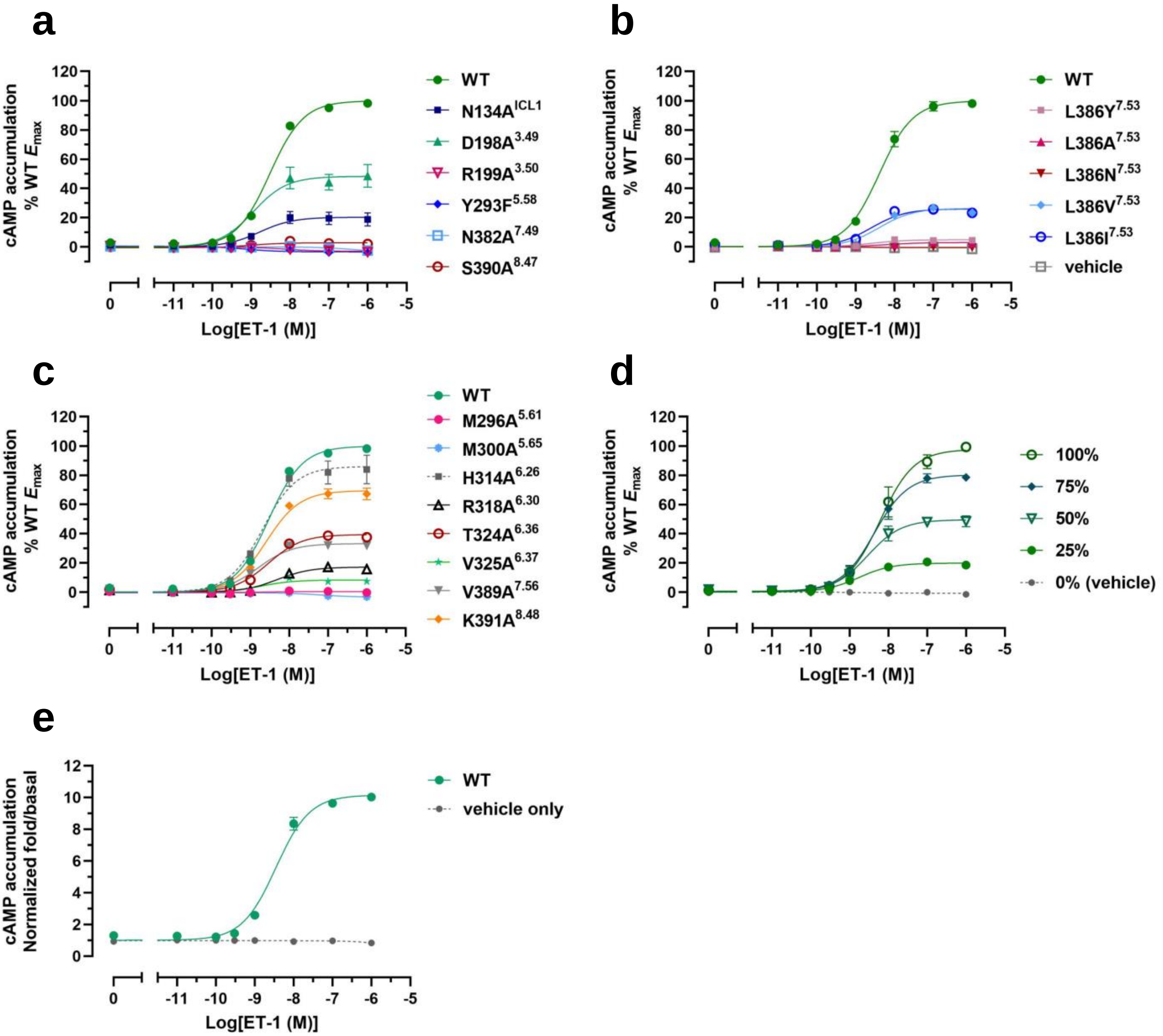

Supplementary Fig. 10: ET-1-induced activation of WT and mutant ET_B receptors in the G_s-mediated cAMP accumulation assay. (a–c) G_s-mediated cAMP accumulation activities of mutant receptors. Three independent experiments were performed in duplicate. **(d)** Signaling of reduced levels of WT ET_BR (%DNA transfected) for G_s is presented. WT (100%) corresponds to the transfection of 500 ng of ET_BR expression plasmid for one well of a 6-well plate in the cAMP accumulation assay, as described in the Methods section. The signals demonstrate a nearly proportional relationship with the amount of expressed receptors in the cAMP accumulation assays. **(e)** cAMP accumulation with increasing concentrations of ET-1 in the WT (100%) and vehicle transfected cells are represented as normalized fold/basal.

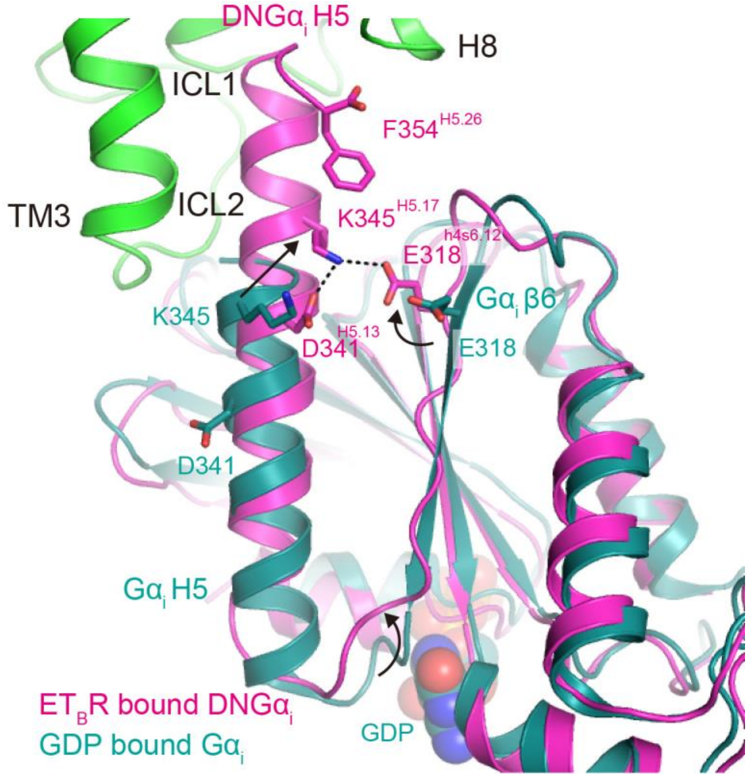

Supplementary Fig. 11: Intramolecular interactions surrounding helix H5 of Gα_i. TM5 and 6 of ET_BR are omitted for clarity. The translation and twist of helix H5 of Gα_i during coupling with ET_BR resulted in K345$^{H5.17}$ interacting with F354$^{H5.26}$, D341$^{H5.13}$, and E318$^{h4s6.12}$ within Gα_i.

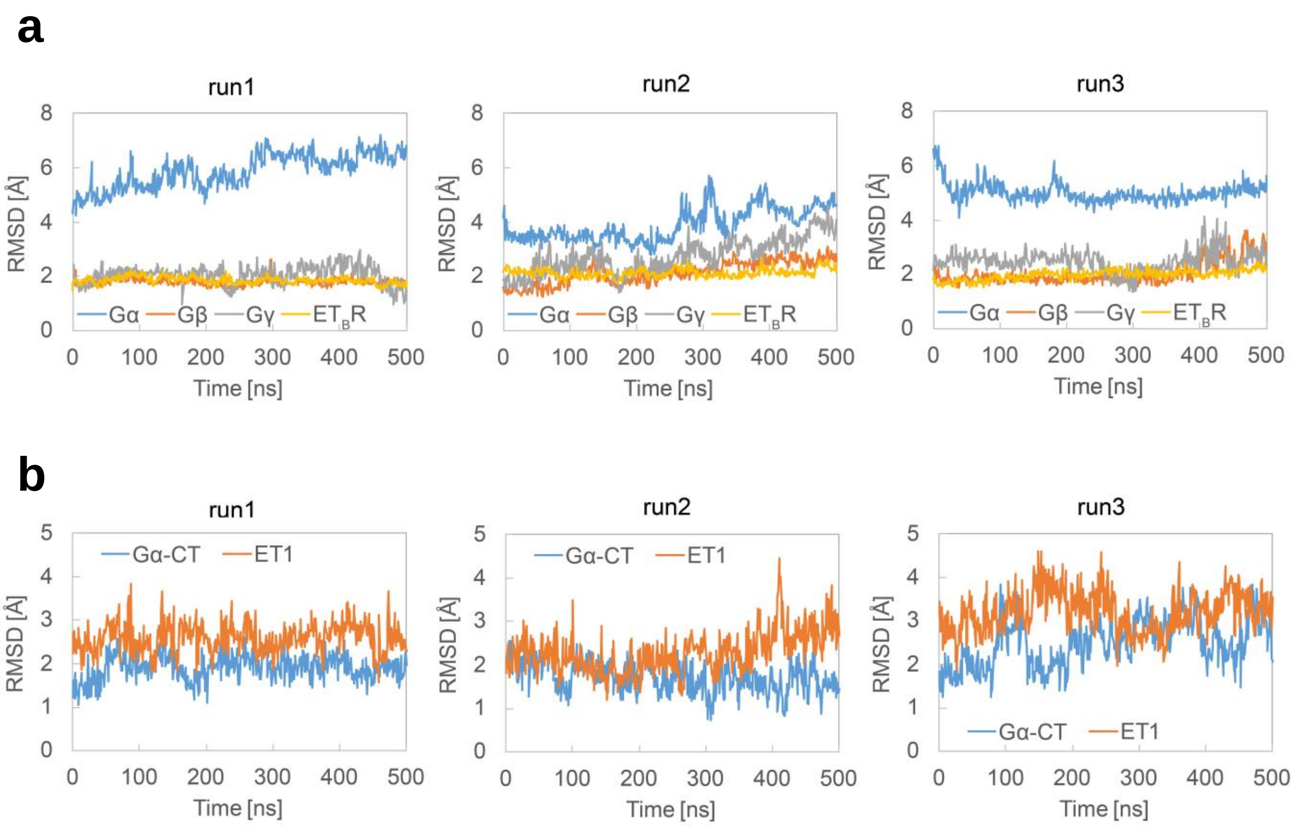

Supplementary Fig. 12: Time evolution of Cα RMSDs. The time evolution of Cα RMSDs is presented in two context types. **(a)** The Cα RMSDs of ET$_B$R, Gα_i, Gβ, and Gγ are tracked relative to their initial structures. **(b)** The Cα RMSDs of ET-1 and C-terminal α5 helix of Gα_i (residues 335–354) are monitored following the superposition of Cα atoms of ET$_B$R onto the initial structure.

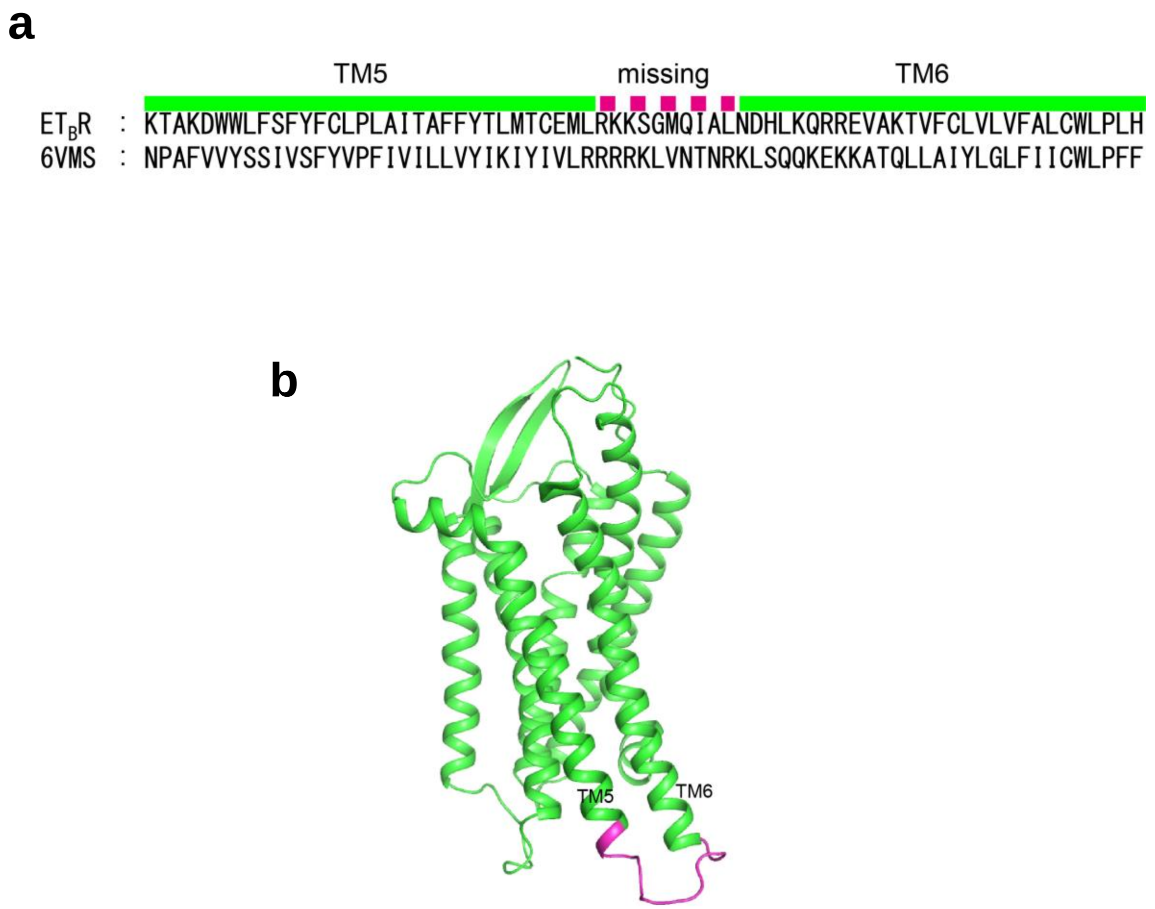

Supplementary Fig. 13: Structural modeling of the missing region between TM5 and TM6. (a) Sequence alignment of ET_BR and D2 dopamine receptor (PDB ID: 6VMS). The resolved and unresolved region are shown as a green line and magenta dashed line, respectively. **(b)** The structure used in MD is colored as shown in (a). The intracellular loop between TM 5 and TM6 of D2 dopamine receptor was used as a template for the missing region of ET_BR.

Supplementary References

1. J. Agirre, M. Atanasova, H. Bagdonas, C.B. Ballard, A. Baslé, J. Beilsten-Edmands, R.J. Borges, D.G. Brown, J.J. Burgos-Mármol, J.M. Berrisford, P.S. Bond, I. Caballero, L. Catapano, G. Chojnowski, A.G. Cook, K.D. Cowtan, T.I. Croll, J.É. Debreczeni, N.E. Devenish, E.J. Dodson, T.R. Drevon, P. Emsley, G. Evans, P.R. Evans, M. Fando, J. Foadi, L. Fuentes-Montero, E.F. Garman, M. Gerstel, R.J. Gildea, K. Hatti, M.L. Hekkelman, P. Heuser, S.W. Hoh, M.A. Hough, H.T. Jenkins, E. Jiménez, The CCP4 suite: integrative software for macromolecular crystallography. *Acta Crystallogr. D Struct. Biol.* 79, 449-461 (2023)

Supplementary Fig. 14: Original uncropped gel images

Fig. S1(a)

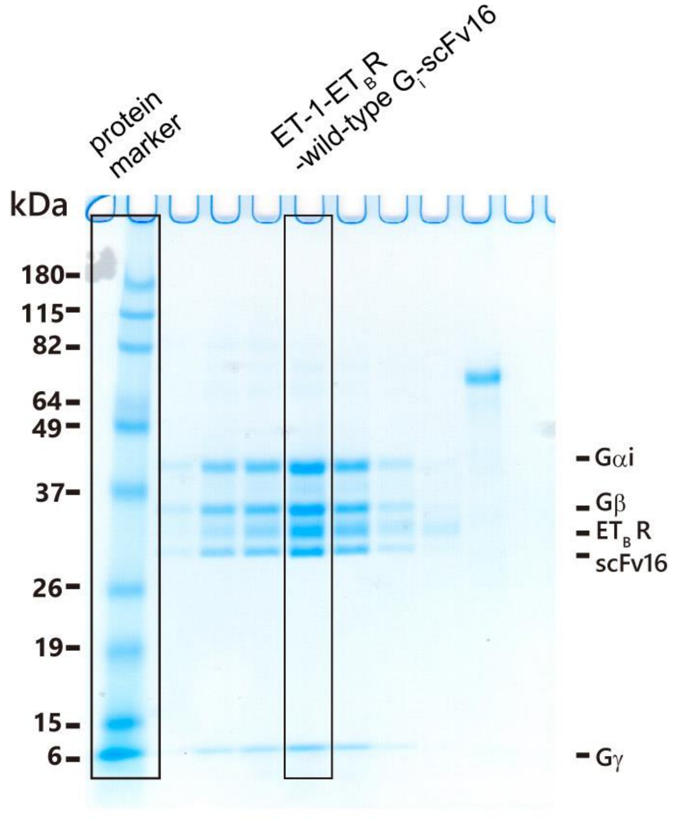

Uncropped gel image

Fig. S2(a)

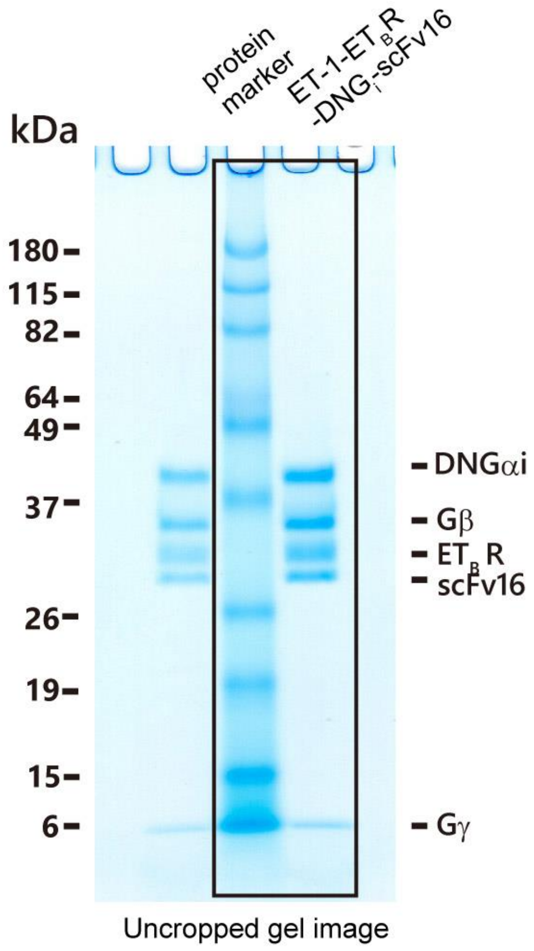
protein marker
ET-1-ET$_B$R -DNG$_i$-scFv16
kDa
180
115
82
64
49
37
26
19
15
6
DNGαi
Gβ
ET$_B$R
scFv16
Gγ
Uncropped gel image
