# Supplementary material for: Structure of endothelin ETB receptor–Gi complex in a conformation stabilized by unique NPxxL motif
Source: Commun Biol. 2024 Oct 16;7:1303. doi: 10.1038/s42003-024-06905-z (PMC11484851; doi:10.1038/s42003-024-06905-z)
Supplement: Supplementary file 5 — Reporting Summary [file 42003_2024_6905_MOESM5_ESM.pdf]

nature portfolio

Corresponding author(s): Kazutoshi Tani and Tomoko Doi

Last updated by author(s): Jun 18, 2024

Reporting Summary

Nature Portfolio wishes to improve the reproducibility of the work that we publish. This form provides structure for consistency and transparency in reporting. For further information on Nature Portfolio policies, see our Editorial Policies and the Editorial Policy Checklist.

Statistics

For all statistical analyses, confirm that the following items are present in the figure legend, table legend, main text, or Methods section.

n/a	Confirmed	
☐	☒	The exact sample size (n) for each experimental group/condition, given as a discrete number and unit of measurement
☐	☒	A statement on whether measurements were taken from distinct samples or whether the same sample was measured repeatedly
☒	☐	The statistical test(s) used AND whether they are one- or two-sided<br>*Only common tests should be described solely by name; describe more complex techniques in the Methods section.*
☒	☐	A description of all covariates tested
☒	☐	A description of any assumptions or corrections, such as tests of normality and adjustment for multiple comparisons
☐	☒	A full description of the statistical parameters including central tendency (e.g. means) or other basic estimates (e.g. regression coefficient) AND variation (e.g. standard deviation) or associated estimates of uncertainty (e.g. confidence intervals)
☒	☐	For null hypothesis testing, the test statistic (e.g. F, t, r) with confidence intervals, effect sizes, degrees of freedom and P value noted<br>*Give P values as exact values whenever suitable.*
☒	☐	For Bayesian analysis, information on the choice of priors and Markov chain Monte Carlo settings
☒	☐	For hierarchical and complex designs, identification of the appropriate level for tests and full reporting of outcomes
☒	☐	Estimates of effect sizes (e.g. Cohen's d, Pearson's r), indicating how they were calculated

Our web collection on statistics for biologists contains articles on many of the points above.

Software and code

Policy information about availability of computer code

Data collection	CryoEM movies are acquired either on the CRYO-ARM at SPring-8 or the Arctica at OIST using Serial EM 3.8 and yoneoLocr 1.0 or EPU 1.20, respectively.
Data analysis	RELION-3.1, CTFFIND-4.1, UCSF Chimera 1.15, UCSF Chimera X 1.4, Phenix 1.20.1, Coot 0.9.6, Pymol 2.5.2, GraphPad Prizm 9

For manuscripts utilizing custom algorithms or software that are central to the research but not yet described in published literature, software must be made available to editors and reviewers. We strongly encourage code deposition in a community repository (e.g. GitHub). See the Nature Portfolio guidelines for submitting code & software for further information.

Data

Policy information about availability of data

All manuscripts must include a data availability statement. This statement should provide the following information, where applicable:

- Accession codes, unique identifiers, or web links for publicly available datasets
- A description of any restrictions on data availability
- For clinical datasets or third party data, please ensure that the statement adheres to our policy

Structure coordinates and density maps have been deposited in the Protein Data Bank (PDB) and the Electron Microscopy Data Bank (EMDB) with accession codes PDB-8XWP and EMDB-38740 for ET-1 bound ETBR-DNGi-scFv16 complex; PDB-8XWQ and EMDB-38741 for ET-1 bound ETBR-wild type Gi-scFv16 complex; PDB-8ZRT and EMD-60404 for the focused 3D refinement of ETBR in the ET-1-bound ETBR–DNG¬i1-scFv16 complex.

Research involving human participants, their data, or biological material

Policy information about studies with human participants or human data. See also policy information about sex, gender (identity/presentation), and sexual orientation and race, ethnicity and racism.

Reporting on sex and gender	N/A
Reporting on race, ethnicity, or other socially relevant groupings	N/A
Population characteristics	N/A
Recruitment	N/A
Ethics oversight	N/A

Note that full information on the approval of the study protocol must also be provided in the manuscript.

Field-specific reporting

Please select the one below that is the best fit for your research. If you are not sure, read the appropriate sections before making your selection.

☒ Life sciences ☐ Behavioural & social sciences ☐ Ecological, evolutionary & environmental sciences

For a reference copy of the document with all sections, see nature.com/documents/nr-reporting-summary-flat.pdf

Life sciences study design

All studies must disclose on these points even when the disclosure is negative.

Sample size	Each dataset comprises an ample number of original movies, from which over a million raw particles were selected using an automated particle-picking program. A total of two datasets were collected to determine two structures, with the number of micrographs and particles for each dataset provided in Supplementary Table 1.
Data exclusions	No data were excluded from the analyses.
Replication	All the functional assay were performed at least three independent experiments. All attempts at replication were succeussful.
Randomization	Randomization was not relevant to this study, because the data collections were automatically.
Blinding	Blinding was unnecessary for the structural and functional experiments since no subjective assignments were involved.

Reporting for specific materials, systems and methods

We require information from authors about some types of materials, experimental systems and methods used in many studies. Here, indicate whether each material, system or method listed is relevant to your study. If you are not sure if a list item applies to your research, read the appropriate section before selecting a response.

Materials & experimental systems

n/a	Involved in the study	
☐	☒	Antibodies
☐	☒	Eukaryotic cell lines
☒	☐	Palaeontology and archaeology
☒	☐	Animals and other organisms
☒	☐	Clinical data
☒	☐	Dual use research of concern
☒	☐	Plants

Methods

n/a	Involved in the study	
☒	☐	ChIP-seq
☒	☐	Flow cytometry
☒	☐	MRI-based neuroimaging

Antibodies

Antibodies used	The single chain antibody scFv16 was used for stabilization of the ETBR-Gi complex and produced in Hi5 cells using a synthetic gene, as originally described by Maeda et al., Nature commun. 9: 3712 (2018). The mouse anti-ETBR monoclonal antibody 2A5 was used for

protein purification purposes by immobolizing on the Sepharose resin.

Validation	The scFv16 sequence was validated by DNA sequencing of the transfer vector, as well as complex formation of the purified scFv16 protein with the heterotrimeric Gi protein. The epitope determination for the 2A5 mAb and the purification procedure of ETBR were described by Yamaguchi et al., Biotech. Lett. 26, 293-296 (2004).

Eukaryotic cell lines

Policy information about cell lines and Sex and Gender in Research

Cell line source(s)	Sf9 (Thermo Fisher Scientific, Cat# B82501), Tni cells (Hi-5) (Expression systems, Cat# 94-011S)<br>HEK293A (Thermo Fisher Scientific, Cat#R70507)
Authentication	Used as expression strains only, independent verification after purchase is not necessary.
Mycoplasma contamination	Cell lines were tested and free from mycoplasma contamination.
Commonly misidentified lines<br>(See ICLAC register)	No commonly misidentified cell lines were used.

Plants

Seed stocks	*Report on the source of all seed stocks or other plant material used. If applicable, state the seed stock centre and catalogue number. If plant specimens were collected from the field, describe the collection location, date and sampling procedures.*
Novel plant genotypes	*Describe the methods by which all novel plant genotypes were produced. This includes those generated by transgenic approaches, gene editing, chemical/radiation-based mutagenesis and hybridization. For transgenic lines, describe the transformation method, the number of independent lines analyzed and the generation upon which experiments were performed. For gene-edited lines, describe the editor used, the endogenous sequence targeted for editing, the targeting guide RNA sequence (if applicable) and how the editor was applied.*
Authentication	*Describe any authentication procedures for each seed stock used or novel genotype generated. Describe any experiments used to assess the effect of a mutation and, where applicable, how potential secondary effects (e.g. second site T-DNA insertions, mosiacism, off-target gene editing) were examined.*
