# Supplementary material for: Structure of endothelin ETB receptor–Gi complex in a conformation stabilized by unique NPxxL motif
Source: Commun Biol. 2024 Oct 16;7:1303. doi: 10.1038/s42003-024-06905-z (PMC11484851; doi:10.1038/s42003-024-06905-z)
Supplement: Supplementary file 1 — Peer review file [file 42003_2024_6905_MOESM1_ESM.pdf]

nature portfolio

Peer Review File

Structure of endothelin ETB receptor–Gi complex in a conformation stabilized by unique NPxxL motif

Open Access This file is licensed under a Creative Commons Attribution 4.0 International License, which permits use, sharing, adaptation, distribution and reproduction in any medium or format, as long as you give appropriate credit to the original author(s) and the source, provide a link to the Creative Commons license, and indicate if changes were made. In the cases where the authors are anonymous, such as is the case for the reports of anonymous peer reviewers, author attribution should be to 'Anonymous Referee' followed by a clear attribution to the source work. The images or other third party material in this file are included in the article's Creative Commons license, unless indicated otherwise in a credit line to the material. If material is not included in the article's Creative Commons license and your intended use is not permitted by statutory regulation or exceeds the permitted use, you will need to obtain permission directly from the copyright holder. To view a copy of this license, visit http://creativecommons.org/licenses/by/4.0/.

Reviewers' comments:

Reviewer #1 (Remarks to the Author):

In this manuscript, Tani et al reported the cryo-EM structure of endothelin ETB receptor–Gi complex. Notably, the complex formation is mediated through a NPXXL motif instead of a NPXXY motif. Combined with MD simulations and mutagenesis studies, the manuscript reveals the essential roles of the NPXXL motif in stabilizing the active conformation of the ETBR receptor.
The electron density map for the receptor part is not perfect. However, the electron densities for the key residues such as the NPXXL motif are acceptable. Nevertheless, I would like to suggest the authors to re-process the data. It seems that the alignment center for the complex is now at the G protein region. As a result, the G protein part has the highest resolution. The authors may try to adjust the alignment center to the receptor part and try to improve the electron density for the receptor.
Other suggestions include:
1, In the structure, the distance between L1953.46 and L3867.53 is approximately 7.3 Å. As a result, there is no direct interaction between these two residues, instead, the interaction is mediated through I1402.43. However, some of the sentences in the manuscript are sort of misleading. For example:
Line 133-134: L3867.53 formed a series of hydrophobic interactions through I1402.43 and L1953.46 to stabilize helical contacts between TM2, TM3, and TM7.
Line 149-150: L3867.53 stabilized this active conformation through a series of hydrophobic interactions with I1402.43 and L1953.46.
When I read these sentences, my first impression was that L3867.53 formed direct hydrophobic interaction with L1953.46. The authors may consider adjust these sentences to be more accurate.
2, Line 105-106: Both refined models are nearly identical with an RMSD value of 0.662 Å. The authors should specify if the RMSD values are calculated from all atoms or Cα atoms.
3, The authors used cAMP Glo-sensor assay to check the effects of the mutations. However, ETBR activates both Gs and Gi, which have opposite effects on cAMP accumulation. The authors need to explain why ETBR activation results in increased cAMP concentration in the assay.
4, Some of the sentences in the manuscript are redundant. For example, line 181-182, “the interactions between ETBR and Gi were exclusively mediated through the α5 helix of Gαi”; line 237-238, “Interactions between ETBR and Gi in the complex were exclusively mediated through
the α5 helix of Gαi.” Actually, the entire parts of “ETBR–Gi interface” and “Structural features of the C-terminus of Gαi” described some similar contents. The authors should consider adjust these parts.

Reviewer #2 (Remarks to the Author):

The manuscript by Tani and Maki-Yonekura et al., entitled “Structure of endothelin ETB receptor-Gi complex in a conformation stabilized by a unique NPxxL motif,” provides structural insights into the role of the rarely observed NPxxL motif in receptor activation and G protein interaction. The authors determined the structure of ET1-bound ETBR-Gi (wild type) first, which resulted in a lower resolution 3D reconstruction of the structure. By using a dominant negative G protein variant, they have successfully elucidated the structure of the same complex at 3.2 Å. The authors present an extensive structural analysis of the interaction interfaces between complex components. After deep structural analysis, the authors provide insights into the contributions of the Leu7.53 residue in the NPxxL motif for receptor activation and G protein interaction and activation, which is the novelty of the study. After careful reading of the manuscript with great interest, I recommend its publication in the journal Communications Biology. I have a short list of minor revisions for the authors to make before its acceptance.

I would like the authors to pay attention and make the following changes;

1. It would have been better to label components of the structure in Fig. 1b and the resolution in the figure. Also, the density of the ligand in an inset panel.

2. As the structure with higher resolution is primarily used for structure analysis, the cryo-EM map and corresponding model should be provided in any of the figure panels before discussing the structural features. The figure panel should display the resolution and the EM density of the ligand.

3. It would make more sense to provide the corresponding EM densities for the illustrated residues that are involved in interaction networks, at least in the main figure panels. For example, Fig. 2b-e, 3a-b, critical residues in 4a.

4. I would highly recommend the authors come up with a better representation of the panel in Fig. 4a. It looks very clumsy and difficult to comprehend.

5. It would be great to provide the EM densities for the water molecules that the authors suspect is contributing to the interaction network displayed in panel Fig. 5b.

6. Spelling mistakes in Supplementary Fig. 1d (subtaction) and Fig. 2d (subtraction and refinment).

Reviewer #3 (Remarks to the Author):

Structure of endothelin ETB receptor–Gi complex …….unique NPxxL motif
Tani et al. (Tomoko Doi, corresponding au.)
Summary of the work: In this manuscript, the authors determined the crystal structures of a thermostabilized ETBR. Structures of ET-1- bound, ligand-free, and antagonist bosentan-bound states are solved by cro-EM studies. Although the ET-1-bound ETBR structure detailed the binding of ET-1 to the receptor, it did not explain the activation mechanism due to lysozyme fusion in the ECL3. Current work was designed to understand ETBR activation by ET-1 and its coupling with G proteins. Thus, the structure of the ET-1-bound ETBR–Gi complex is reported by using cryo-electron microscopy (cryo-EM), and further evaluated with MD simulations and mutagenesis studies.
I can not comment on the quality of the structural data and computational analysis that generated the models presented. Please refer to experts in this area.
The authors identified a unique feature—the downward motion of TM7 during activation through a non-canonical NPxxL motif, which leads to the formation of a hydrophobic binding pocket for the C-terminal α5 helix of Gαi. They performed site-directed mutagenesis to validate the hydrophobic interaction that stabilizes ETBR-Gi interaction.
The mutagenesis and functional data presented are well organized and support the interpretation that the non-canonical NPxxL motif engages Gi by creating a hydrophobic pocket for Gi to bind.
Minor weakness I see is that the data presented is highly technical. But the work is very relevant for experts on the mechanism of GPCR activation demonstrating a novel mechanism.
Overall, the manuscript is well written, and the presentation of data in the figures is clear.

Response to reviewers:

Reviewer #1

Reviewer #1's comments:
In this manuscript, Tani et al reported the cryo-EM structure of endothelin ETB receptor–Gi complex. Notably, the complex formation is mediated through a NPXXL motif instead of a NPXXY motif. Combined with MD simulations and mutagenesis studies, the manuscript reveals the essential roles of the NPXXL motif in stabilizing the active conformation of the ETBR receptor. The electron density map for the receptor part is not perfect. However, the electron densities for the key residues such as the NPXXL motif are acceptable. Nevertheless, I would like to suggest the authors to re-process the data. It seems that the alignment center for the complex is now at the G protein region. As a result, the G protein part has the highest resolution. The authors may try to adjust the alignment center to the receptor part and try to improve the electron density for the receptor.

Other suggestions include:
1, In the structure, the distance between L195$^{3.46}$ and L386$^{7.53}$ is approximately 7.3 Å. As a result, there is no direct interaction between these two residues, instead, the interaction is mediated through I140$^{2.43}$. However, some of the sentences in the manuscript are sort of misleading. For example:
Line 133-134: L386$^{7.53}$ formed a series of hydrophobic interactions through I140$^{2.43}$ and L195$^{3.46}$ to stabilize helical contacts between TM2, TM3, and TM7.
Line 149-150: L386$^{7.53}$ stabilized this active conformation through a series of hydrophobic interactions with I140$^{2.43}$ and L195$^{3.46}$. When I read these sentences, my first impression was that L386$^{7.53}$ formed direct hydrophobic interaction with L195$^{3.46}$. The authors may consider adjust these sentences to be more accurate.

2, Line 105-106: Both refined models are nearly identical with an RMSD value of 0.662 Å. The authors should specify if the RMSD values are calculated from all atoms or Cα atoms.

3, *The authors used cAMP Glo-sensor assay to check the effects of the mutations.*

However, ET_BR activates both Gs and Gi, which have opposite effects on cAMP accumulation. The authors need to explain why ET_BR activation results in increased cAMP concentration in the assay.

4, Some of the sentences in the manuscript are redundant. For example, line 181-182, "the interactions between ET_BR and Gi were exclusively mediated through the α5 helix of Gαi"; line 237-238, "Interactions between ET_BR and G_i in the complex were exclusively mediated through the α5 helix of Gαi." Actually, the entire parts of "ETBR–Gi interface" and "Structural features of the C-terminus of Gαi" described some similar contents. The authors should consider adjust these parts.

Our response:

We thank the reviewer for their suggestions and support of our work. Following the reviewer's recommendation, we conducted focused 3D refinement to obtain receptor densities by adjusting the alignment center to the receptor. The focused refined map slightly improved the local resolution compared with the whole complex map, as expected by reviewer #1 (Supplementary Fig. 5). Both structures are nearly identical; however, the focused refined map near the Gα docking region was largely disordered. Owing to the high similarity of these structures (Supplementary Fig. 6b), we decided to use the whole complex map as the reference in the text. We have included the following sentences and made modifications to Supplementary Figs. 2 and 6. In addition, we have deposited the focused 3D refinement map and its model as EMDB-60404 and PDB-8ZRT, respectively (Supplementary Table S1).

Lines 105–113: Furthermore, we performed focused 3D refinement to obtain receptor densities at a resolution of 3.6 Å. Receptor density was assessed in the ET_BR–DNG_{i1} complex after adjusting the alignment center to the receptor (Supplementary Figs. 2, 5, Table 1). Both ET_BR–G_i complex models are nearly identical—their Cα atoms have an RMSD of 0.662 Å (Supplementary Fig. 6a). Compared with the ET-1 bound ET_BR model in ET_BR–DNG_{i1}, the small RMSD values of the Cα atoms and the similar residue conformations in the other two models indicate they are nearly identical (0.391 Å for ET_BR–wild-type G_{i1} and 0.364 Å for the focused 3D refinement of ET_BR) (Supplementary Fig. 6b, c).

Lines 494–497: Particle projections were subjected to subtraction of the detergent micelle density followed by 3D auto-refinement, yielding a final map with resolutions of 4.61, 3.21, and 3.62 Å for ET_BR–WTG_i, ET_BR–DNG_i, and ET_BR after focused 3D classification, respectively…

<Response to suggestion 1>
As suggested, we have corrected the sentences as follows:

Lines 140–142: $L386^{7.53}$ formed a hydrophobic interaction with $I140^{2.43}$ to stabilize the helical contacts between TM2 and TM7.

Lines 156–159: The downward shift of TM7 was stabilized by a hydrophobic interaction between $L386^{7.53}$ and $I140^{2.43}$, which simultaneously interacted with $L195^{3.46}$ (Fig. 3a). Despite the considerable distances between residues 3.46 and 7.53, precluding direct contacts, this conformation could be maintained.

<Response to suggestion 2>
The calculation of RMSD was performed using Cα atoms. We have edited the main text as follows:

Line 109: their $C\alpha$ atoms have an RMSD of 0.662 Å

<Response to suggestion 3>
As indicated by reviewer #1, the GloSensor cAMP assay detects changes in intracellular cAMP concentration, primarily resulting from the stimulation or inhibition of adenylyl cyclase (AC) via G_s or $G_{i/o}$ proteins, respectively, following GPCR activation. In our experimental setup, the transient overexpression of the promiscuous ET_B receptor in HEK293 cells and the subsequent activation of the receptor indicate that signals are mediated by endogenous G proteins in HEK293 cells. Under our experimental conditions, luminescence was measured 5 min after agonist addition at room temperature, and significant fold inductions were observed without the addition of phosphodiesterase inhibitors or pertussis toxin, which inhibits $G_{i/o}$ proteins (Fig. 1). This indicates that in HEK293 cells, the signals mediated by G_s proteins were notably strong and dominated $G_{i/o}$ responses within

this time frame.

One plausible reason for this observation is the high level of endogenous G_s protein in HEK293 cells, although the mechanism by which AC activity is inhibited is unclear. Another possibility is that ET_BR selectivity favors G_s, which contrasts with the typical behavior of promiscuous ET_BRs based on the systematic screening of selective GPCR–G protein couplings (Inoue et al., 2019; Sandhu et al., 2022). A study (Fig. 2 of Lane, J.R. et al., 2008) has shown that treatment with pertussis toxin to inactivate $G_{i/o}$ in HEK293T* cells expressing G_i-coupled dopamine D2 and D3 receptors did not considerably alter the basal activity of G_i, suggesting low levels of endogenous $G_{i/o}$ in HEK293 cells.

Preliminary results from our investigation into the expression of $G\alpha_s$ and $G\alpha_{i1}$ subunits fused with the large fragment of Nanobit luciferase in HEK293A cells are shown in Fig. 2. These expression plasmids were prepared for the G protein dissociation assay described in the Methods and Materials section. Following transfection with pcDNA3.1-based plasmids encoding these fusion proteins, cell extracts were analyzed by immunoblotting using anti-$G\alpha_{s/olf}$ (C-18) (Santa Cruz) and anti-rat $G\alpha_i$ (Upstate Biotech Inc.) antibodies, as required. Distinct bands were observed for $G\alpha_s$-Lg and $G\alpha_i$-Lg at ~65 kDa (indicated by blue arrows), in addition to bands for endogenous $G\alpha_s$ and $G\alpha_i$ at approximately 40–45 kDa (indicated by red arrowheads), derived from HEK293A cells. Although the $G\alpha_s$ blot was somewhat disordered (lanes 1 and 2), the band for endogenous $G\alpha_s$ was distinct, whereas that for endogenous $G\alpha_i$ was faint (lanes 3 and 4). Comparison of band intensities between endogenous Gα and transiently expressed Gα-Lg revealed that the intensity of endogenous $G\alpha_i$ was notably lower, whereas that of endogenous $G\alpha_s$ was comparable with that of $G\alpha_s$-Lg.

Therefore, we hypothesize that the higher level of $G\alpha_s$ than $G\alpha_i$ in HEK293 cells contributed to the increase in cAMP concentration upon ET_BR activation. We have included additional details in the Materials and Methods section (GloSensor cAMP assay; lines 597–598) and included Fig. 1 as Supplementary Figure 10e to illustrate the ET_BR-mediated increase in G_s responses in HEK293 cells.

*) The HEK293T cell line is a variant of HEK293 that contains an SV40 large T antigen mutant gene. The HEK293A cell line used in this study is a subclone of

the parent HEK293 cells with a relatively flat morphology. The levels of various Gα proteins expressed are similar in all three cell lines.

1) Inoue, A. et al. Illuminating G-Protein-Coupling Selectivity of GPCRs. *Cell* **177**, 1933–1947 (2019)
2) Sandhu, M. et al., Dynamic spatiotemporal determinants modulate GPCR:G protein coupling selectivity and promiscuity. Nature Communi. 13:7428 (2022)
3) Lane, J.R. et al., G Protein Coupling and Ligand Selectivity of the D_{2L} and D_3 Dopamine Receptors. J. Pharmacol. Exp. Ther. 325, 319-330 (2008).

Fig. 1

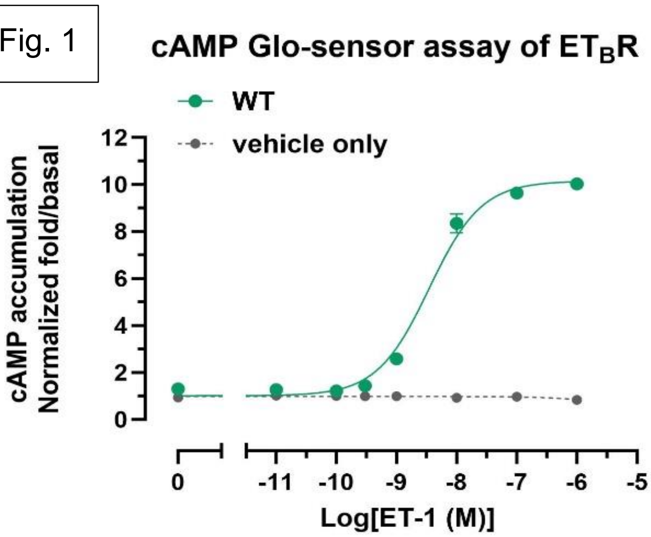

Fig. 2

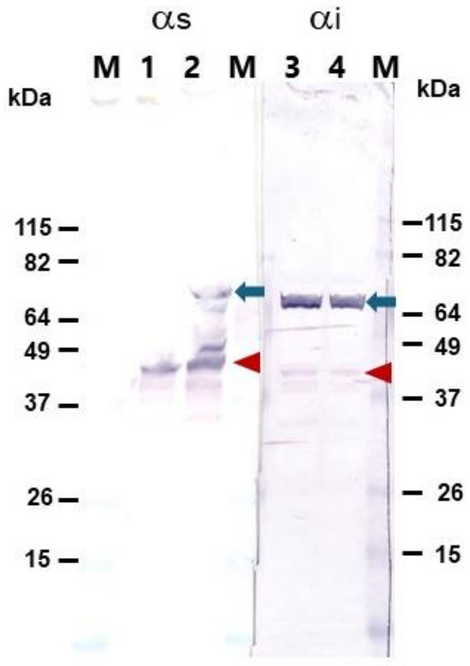

Lane 1: 4 μL of 50 μL untransfected cell extract.
Lane 2: 4 μL of 50 μL Gαs-Lg transfected cell extract.
Lane 3: 4 μL of 50 μL Gαi-Lg transfected cell extract.
Lane 4: 2 μL of 50 μL Gαi-Lg transfected cell extract.
M: molecular weight marker.

<Response to suggestion 4>

Following recommendations to revise two sections, we have divided three sections to improve readability as follows:

1. The “ET_BR–Gi interface” section explains observed interactions based on the structure.
2. The “ET_BR–Gi dissociation assay” section describes functional experiments related to the ET_BR–Gi interface.
3. The “ET_BR coupled through the C-terminus of Gα” section focuses on the features of ET_BR interactions as a promiscuous coupler and the C-terminus of Gα.

In accordance with these section modifications, we have deleted redundant

sentences as suggested.

Lines 210–214: The sentence “Interactions with ET_BR were …” has been deleted.

Lines 237 and 238: The sentence “Interactions between ET_BR and Gi …” has been deleted.

Reviewer #2

<u>Reviewer #2’s comments</u>:

The manuscript by Tani and Maki-Yonekura et al., entitled “Structure of endothelin ETB receptor-Gi complex in a conformation stabilized by a unique NPxxL motif,” provides structural insights into the role of the rarely observed NPxxL motif in receptor activation and G protein interaction. The authors determined the structure of ET1-bound ETBR-Gi (wild type) first, which resulted in a lower resolution 3D reconstruction of the structure. By using a dominant negative G protein variant, they have successfully elucidated the structure of the same complex at 3.2 Å. The authors present an extensive structural analysis of the interaction interfaces between complex components. After deep structural analysis, the authors provide insights into the contributions of the Leu7.53 residue in the NPxxL motif for receptor activation and G protein interaction and activation, which is the novelty of the study. After careful reading of the manuscript with great interest, I recommend its publication in the journal Communications Biology. I have a short list of minor revisions for the authors to make before its acceptance.

I would like the authors to pay attention and make the following changes;

1. It would have been better to label components of the structure in Fig. 1b and the resolution in the figure. Also, the density of the ligand in an inset panel.

2. As the structure with higher resolution is primarily used for structure analysis, the cryo-EM map and corresponding model should be provided in any of the figure panels before discussing the structural features. The figure panel should display the resolution and the EM density of the ligand.

3. It would make more sense to provide the corresponding EM densities for the illustrated residues that are involved in interaction networks, at least in the main figure panels. For example, Fig. 2b-e, 3a-b, critical residues in 4a.

4. I would highly recommend the authors come up with a better representation of the panel in Fig. 4a. It looks very clumsy and difficult to comprehend.

5. It would be great to provide the EM densities for the water molecules that the authors suspect is contributing to the interaction network displayed in panel Fig. 5b.

6. Spelling mistakes in Supplementary Fig. 1d (subtaction) and Fig. 2d (subtraction and refinment).

Our response:

We thank Reviewer #2 for the positive assessment of our work. To address the reviewer's suggestions, we have made the following changes in the revised manuscript:

<Response to suggestion 1>

The names of structural components and map resolution are labeled in Fig. 1b and 1a, respectively. In addition, the ET-1 structure with its density map is shown in the inset of Fig. 1a.

<Response to suggestion 2>

We believe that our revision improved based on suggestion 1 now aligned to this comment.

<Response to suggestion 3>

Following suggestions 3 and 4, we have selected Fig. 3a and c to represent cryo-EM density. In Fig. 3c, density corresponding only to the residues of H5 of Gαi is depicted to enhance clarity and recognition.

<Response to suggestion 4>
Based on this suggestion, we have divided the old Fig. 4 into new Figs. 4 and 5, presenting structural and biochemical data, respectively. This modification also addresses suggestion 4 from reviewer #1.

<Response to suggestion 5>
We appreciate this comment, because the current resolution of 3.2 Å is not sufficient to observe water molecules. However, we hypothesize that the density around the tip of Arg199$^{3.50}$ can be assigned as water density, contributing to the hydrogen network in the cavity. In Fig. 3a, we have added an arrowhead to indicate water density. In addition, we have inserted the following sentence in the main text:

Lines 307–309: Accordingly, a relatively bulky density at the tip of R199$^{3.50}$ observed in the cryo-EM map can be attributed to water, contributing to the network (an arrowhead in Fig. 3a).

<Response to suggestion 6>
The spellings in Supplementary Figs. 1d and 2d have been corrected.

Reviewer #3

__**Reviewer #3's comments**__**:**
Structure of endothelin ETB receptor–Gi complex …….unique NPxxL motif Tani et al. (Tomoko Doi, corresponding au.)
Summary of the work: In this manuscript, the authors determined the crystal structures of a thermostabilized ETBR. Structures of ET-1- bound, ligand-free, and antagonist bosentan-bound states are solved by cro-EM studies. Although the ET-1-bound ETBR structure detailed the binding of ET-1 to the receptor, it did not explain the activation mechanism due to lysozyme fusion in the ECL3. Current work was designed to understand ETBR activation by ET-1 and its coupling with G proteins. Thus, the structure of the ET-1-bound ETBR–Gi complex is reported by using cryo-electron microscopy (cryo-EM), and further

evaluated with MD simulations and mutagenesis studies.
I can not comment on the quality of the structural data and computational analysis that generated the models presented. Please refer to experts in this area.
The authors identified a unique feature—the downward motion of TM7 during activation through a non-canonical NPxxL motif, which leads to the formation of a hydrophobic binding pocket for the C-terminal α5 helix of Gαi. They performed site-directed mutagenesis to validate the hydrophobic interaction that stabilizes ETBR-Gi interaction. The mutagenesis and functional data presented are well organized and support the interpretation that the non-canonical NPxxL motif engages Gi by creating a hydrophobic pocket for Gi to bind.
Minor weakness I see is that the data presented is highly technical. But the work is very relevant for experts on the mechanism of GPCR activation demonstrating a novel mechanism.
Overall, the manuscript is well written, and the presentation of data in the figures is clear.

Our response:

We appreciate reviewer #3's strong support of our manuscript and hope that the revisions made in response to reviewers #1 and #2 will address any minor weaknesses noted.

We believe these revisions have considerably strengthened our manuscript and hope that our manuscript meets the reviewers' expectations. We appreciate the valuable feedback and trust that the revised manuscript is now suitable for publication.

REVIEWERS' COMMENTS:

Reviewer #1 (Remarks to the Author):

The authors have addressed all my questions.

Reviewer #2 (Remarks to the Author):

The authors have addressed my previously raised concerns.

Reviewer #3 (Remarks to the Author):

Revised manuscript provides RMSD information for the main chain and sidechains, which reasonably addresses my critique.
